# Supplementary material for: Mode of birth and risk of infection-related hospitalisation in childhood: A population cohort study of 7.17 million births from 4 high-income countries
Source: PLoS Med. 2020 Nov 19;17(11):e1003429. doi: 10.1371/journal.pmed.1003429 (PMC7676705; doi:10.1371/journal.pmed.1003429)
Supplement: S1 Analysis Plan — (DOCX) [file pmed.1003429.s002.docx]

**S1 Analysis Plan – Analysis plan for study populations**

**Study population**:

- Live-born, non-adopted, singleton births
- Born between – TBD, based on available dates of data
- Exclusions: children with congenital malformations (ICD-10: Q00-Q99)
- Children followed from *birth-related hospital discharge date* (note: this is not the actual date of birth) to:
  - [**1^st^ OCCURRENCE ANALYSIS]**
    - 1^st^ recorded infection-related hospitalisation (admission), death, emigration, 5^th^ birthday [depends on number of years of available data], or end of study date (TBD, based on available dates of data), whichever occurs first.
  - [**MULTIPLE EVENTS ANALYSIS]**
    - Definition of multiple events = 1^st^, 2^nd^, 3^rd^ infection-related hospitalisations during the study period
    - Death, emigration, 5^th^ birthday, or end of study date (TBD, based on available dates of data), whichever occurs first.

**Exposure groups**:

- Vaginal deliveries
- All caesarean sections
- Emergency caesarean
- Elective caesarean

Each dataset has its own way of identifying mode of delivery so whichever code or way of identification is most accurate should be used.

**Years of data analysis**: exact years TBD based on available data from datasets

Please provide available dates of data for each dataset

- NSW Birth (2001-2012) Hospital (2001-2012)
- WA Birth (1996-2012) Hospital (1996-June 30, 2013)
- England Birth (April 1 1998-Mar 31 2012) Hospital (April 1 1998-Mar 31 2012)
- Scotland Birth (2001-2015) Hospital (2001-2015)
- Denmark Birth (1995-2015?) Hospital (1995-2015?)

**Outcome - infection-related hospitalisations**:

- **Definition** - Has one of the diagnostic ICD 10 codes, either as a primary or secondary diagnosis, at least 24 hours/1day following discharge for the birth-related hospitalisation during the study period.

Coding of outcome:

1. ***Infection-related hospitalisation (looking at presence of any type of IRH):***

- 1^st^ occurrence (admission) of infection-related hospitalisation:
  - Date of onset = 1^st^ recorded date of hospitalisation
  - *Analysis: Cox model for 1^st^ event*
- Multiple events: Infection-related hospitalisations (1^st^, 2^nd^, 3^rd^) during the study period
  - Date of onset = recorded date of contact with the hospital when the patient was hospitalised. Use date of onset for each hospitalisation up until 3^rd^ hospitalisation during the study period.
  - Inter-hospital transfers and repeat hospitalisations within 7 days to be considered as a single admission.
  - Ordered events
    - Each time interval starts at time of previous event
  - [Note: for multiple events analysis the data need to be organised in the long format (i.e. multiple records per person).]
  - Multiple events analysis requires a variable to indicate the number of previous events (0, 1, 2, 3).

Example:

| Date | IRH (any type) | # of previous events |
| --- | --- | --- |
| Sept 1 2010 | 1st | 0 |
| Oct 1 2010 | 2nd | 1 |
| Jan 1 2012 | 3rd | 2 |

- - *Analysis: Cox model for multiple events (eg. Conditional model PWP)*

1. ***Clinical infection subgroups:***

- Invasive bacterial, gastrointestinal, lower respiratory tract, skin and soft tissue, upper respiratory tract, genitourinary, and viral infections, as was defined *a priori*
- 1^st^ occurrence (admission) of infection-related hospitalisation:
  - Type of clinical infection subgroup of 1^st^ admission of infection-related hospitalisation

Example:

| Date | IRH (type) | ~~# of previous events~~ |
| --- | --- | --- |
| Sept 1 2010 | Bacterial | ~~0~~ |
| Oct 1 2010 | Viral | ~~1~~ |
| Jan 1 2012 | Viral | ~~2~~ |

For this analysis, we are only concerned with the first line of data which is a bacterial infection admission on Sept 1, 2010. The # of previous events variable is not used for this particular analysis since we are only looking at the 1^st^ occurrence/event.

- - *Analysis: Cox model for 1^st^ event*
  - Multiple events: Number of infections (1, 2, 3) within each clinical infection subgroup

Example: During the study period a child is first admitted to the hospital for a bacterial infection. A month later they are admitted for a viral infection, then >1 year later for another viral infection. For the analysis they will be categorised as:

- - 1^st^ admission analysis = bacterial infection (as indicated in above table, **section 2**)
  - Multiple events analysis = 1 bacterial infection and 2 viral infections
    - For this analysis, only infections of the same clinical subgroup should be included and counted as a “previous admission”. In the below example, bacterial infection does not have a previous admission, but for the 2 viral infections the first viral admission is coded as a “previous admission” for the second viral admission.

| Date | IRH (type) | # of previous events (bacterial) | # of previous events (viral) |
| --- | --- | --- | --- |
| Sept 1 2010 | Bacterial | 0 | - |
| Oct 1 2010 | Viral | - | 0 |
| Jan 1 2012 | Viral | - | 1 |

This patient will contribute to 2 clinical infection subgroup hazard ratios: one for bacterial infections and one for viral infections

- *Analysis: Cox model for multiple events (eg. Conditional model PWP)*

**Potential confounders** (to be included in all adjusted models):

- Sex (indeterminate sex classify as missing)
- Gestational age, weeks
  - - <28, 28-<32, 32-<34, 34-<36, 36-<38, 38-40, >40 no upper/lower limits (Scotland classifies <24 or >43 as missing – this is ok as an alternative)
    - [Note: we decided to use actual values rather than z-scores for consistency across datasets]
- Birth weight z-scores (gestational age- and sex- specific) please derive from your own study population
  - Percentiles: ≤10, >10-25, >25-75 (ref), >75-90, >90
    - no upper/lower limits (Scotland classifies birth weight <500 or >5500 as missing – this is ok as an alternative)
- Smoking during pregnancy
  - Yes/No
- Maternal age at birth, years
  - <20, 20-<25, 25-<30, 30-<35, ≥35
- Parity
  - 0, 1, 2, ≥3
- Socioeconomic status, most use area level deprivation
  - Quintiles?
- Birth year
  - Include each year as own category in model, rather than in 5 year blocks
- Season of birth
  - Spring, Summer, Autumn, Winter
    - based on solstice and equinox dates: 22 March – 21 June / 22 June – 21 Sept / 22 Sept – 21 Dec / 22 Dec – 21 March
- Indication for type of delivery
  - Yes/No based on whether pregnancy has been coded for either:
    - Hypertensive disorders of pregnancy
      - Gestational hypertension, preeclampsia, eclampsia
    - Diabetes mellitus in pregnancy
      - Gestational or pre-existing

**Statistical Analysis:**

- Cox model (hazard ratios and 95% confidence intervals)
  - 1st occurrence of event
  - Multiple events

All potential confounders listed above to be included in all adjusted models.

See tables presented in ‘Suggested Tables_May2018’ [NOTE: not part of supplementary information] spreadsheet for more detail:

1. Overall risk and PROM stratified analysis
   - 1^st^ occurrence and multiple events hazard ratios
   - PROM defined as presence of ICD 10 code: O42 or ICD 9 code: 658.1
2. Labour stratified analysis – **if possible**
   - 1^st^ occurrence and multiple events hazard ratios
   - This will be messy and unstandardised. Code caesarean groups as occurring before/after onset of labour if data are available
3. Clinical infection subgroups
   - 1^st^ occurrence and multiple events hazard ratios
4. Clinical infection subgroups and PROM – **if possible**
   - 1^st^ occurrence hazard ratios
5. Age (risk) periods
   - 1^st^ occurrence hazard ratios
   - Kaplan-Meier survival plot for 5 year follow-up period?
6. Low risk pregnancy sub-group
   - 1^st^ occurrence and multiple events hazard ratios
   - Low risk pregnancies defined as: children born >=37-<=41 weeks gestation, cephalic presentation, birth weight between the 10th and 90th percentiles for gestational age and sex, born to women aged 20–34 years without medical conditions
     - Definition of women without medical conditions:
       - No reported pre-existing or pregnancy-related hypertension (ICD10 codes: I10-I15, O10-O16) or diabetes (ICD10 codes: E10-E14, O24)
