# Supplementary material for: Mode of birth and risk of infection-related hospitalisation in childhood: A population cohort study of 7.17 million births from 4 high-income countries
Source: PLoS Med. 2020 Nov 19;17(11):e1003429. doi: 10.1371/journal.pmed.1003429 (PMC7676705; doi:10.1371/journal.pmed.1003429)
Supplement: S1 Fig — (DOCX) [file pmed.1003429.s003.docx]

**S1 Fig - Flow charts for site-specific study populations**

| **Denmark** |  | **Scotland** |  | **England** | |  | | **New South Wales** |  | | **Western Australia** | |  |
| --- | --- | --- | --- | --- | --- | --- | --- | --- | --- | --- | --- | --- | --- |
|  |  |  |  |  | |  | |  |  | |  | |  |
| All liveborn births |  | All in hospital deliveries |  | All mother-infant pairs | |  | | All liveborn births |  | | All liveborn births | |  |
| 1997-2010 |  | 2001-2015 |  | 1 April 1998 - 31 March 2010 | |  | | 2001-2012 |  | | 1996-2012 | |  |
| N=913,657 |  | n = 814,321 |  | n=7,335,218 | |  | | n=1,102,075 |  | | n=469,589 | |  |
|  |  |  |  |  | |  | |  |  | |  | |  |
| Singleton births only |  | Matched to birth record |  | Exclude stillbirths or unknown/unrecorded birth status | |  | | Singleton births only |  | | Singleton births only | |  |
| n=875,598 |  | n = 808,220 |  | n=5,787,983 | |  | | n=1,054,963 |  | | n=455,675 | |  |
|  |  |  |  |  | |  | |  |  | |  | |  |
| Exclude congenital malformations (n=90,301) |  | Single live births |  | Singleton births only | |  | | Exclude congenital malformations (n=108,793) and missing data (n=861) |  | | Exclude congenital malformations (n=18,190) | |  |
| **n=783,082** |  | n = 783,767 |  | n=5,623,414 | |  | | **n=945,309** |  | | n=437,485 | |  |
|  |  |  |  |  | |  | |  |  | |  | |  |
|  |  | Exclude congenital malformations (n = 63,952) |  | Exclude missing gestational age (n=833,558) and/or missing birthweight (n=41,928) | |  | |  |  | | Exclude if records not available in Midwives database (n=543) | |  |
|  |  | n = 719,815 |  | n=4,760,883 | |  | |  |  | | **n=436,942** | |  |
|  |  |  |  |  | |  | |  |  | |  | |  |
|  |  | Exclude unknown method of delivery (n = 190) |  | Exclude gestational age <30 or >43 weeks (n=113,132) and/or birthweight <500 or >5499 grams (n=7,917) | |  | |  |  | |  | |  |
|  |  | **n = 719,625** |  | n=4,641,666 | |  | |  |  | |  | |  |
|  |  |  |  |  | |  | |  |  | |  | |  |
|  |  |  |  | Exclude congenital malformations (n=351,837) | |  | |  |  | |  | |  |
|  |  |  |  | **n=4,289,829** | |  | |  |  | |  | |  |
|  |  |  |  |  |  | |  | | |  | |  | |
