# Supplementary material for: Mode of birth and risk of infection-related hospitalisation in childhood: A population cohort study of 7.17 million births from 4 high-income countries
Source: PLoS Med. 2020 Nov 19;17(11):e1003429. doi: 10.1371/journal.pmed.1003429 (PMC7676705; doi:10.1371/journal.pmed.1003429)
Supplement: S3 Fig — Estimates are from recurrent events models fitted for total time. Models adjusted for sex, gestational age, birth weight z-score, maternal age at birth, parity, area level deprivation, birth year, medical indication for type of delivery, and season of birth. Reference is vaginal births. D+L, DerSimonian and Laird random effects model; I-V, inverse-variance weighted fixed effects model. (DOCX) [file pmed.1003429.s005.docx]

**S3 Fig: Site-specific and meta-analysis hazard ratios for infection-related hospitalisation, not adjusted for smoking during pregnancy**

Estimates are from recurrent events models fitted for total time. Models adjusted for: sex, gestational age, birth weight z-score, maternal age at birth, parity, area level deprivation, birth year, medical indication for type of delivery, and season of birth. Reference is vaginal births. I-V: inverse-variance weighted fixed effects model, D+L: DerSimonian and Laird random effects model.
