# Supplementary material for: Mode of birth and risk of infection-related hospitalisation in childhood: A population cohort study of 7.17 million births from 4 high-income countries
Source: PLoS Med. 2020 Nov 19;17(11):e1003429. doi: 10.1371/journal.pmed.1003429 (PMC7676705; doi:10.1371/journal.pmed.1003429)
Supplement: S1 Table — (DOCX) [file pmed.1003429.s006.docx]

**S1 Table – Variable definition**

| **DENMARK** |  |  |
| --- | --- | --- |
| **Description** | **Variable** | **Data source** |
| **Caesarean section** |  |  |
| Emergency CS prelabour | KMCA10A | Medical Birth Register |
| Emergency CS in labour | KMCA10E | Medical Birth Register |
| Elective CS prelabour | KMCA10B | Medical Birth Register |
| Elective CS in labour | KMCA10D | Medical Birth Register |
| **Medical indication for type of delivery** |  |  |
| Hypertensive disorders of pregnancy  (gestational hypertension, preeclampsia, eclampsia) | If following variables contained any of the ICD10 codes I10-I15, O10-O16: |  |
|  | C_GRAVKOMPL ("Pregnancy complications") C_MS ("Medical diseases") C_KMS ("Complicated medical diseases (Danish Society for Obstetrics and Gynecology definition)") C_PRECL ("Preeclampsia") | Medical Birth Register |
|  | Primary/secondary diagnostic codes | National Hospital Register |
| Diabetes mellitus in pregnancy (gestational or pre-existing) | If following variables contained any of the ICD10 codes E10-E14, O24: |  |
|  | C_GRAVKOMPL ("Pregnancy complications") C_MS ("Medical diseases") C_KMS ("Complicated medical diseases Danish Society for Obstetrics and Gynecology definition") | Medical Birth Register |
|  | Primary/secondary diagnostic codes | National Hospital Register |
|  |  |  |
| **SCOTLAND** |  |  |
| **Description** | **Variable** | **Data source** |
| **Caesearean section** |  |  |
| Emergency | Mode of delivery = 8 | SMR02 Maternity Data |
| Elective | Mode of delivery = 7 | SMR02 Maternity Data |
| **Medical indication** |  |  |
| Hypertensive disorders of pregnancy | ICD10 codes I10-I15, O10-O16 | SMR02 Maternity Data |
| Diabetes | ICD10 codes E10-E14, O24 | SMR02 Maternity Data |
|  |  |  |
| **ENGLAND** |  |  |
| **Description** | **Variable** | **Data source** |
| **Caesearean section** |  |  |
| Emergency | delmeth == "8" \| OPCS code R18 | Hospital Episode Statistics |
| Elective | delmeth == "7" \| OPCS code R17 | Hospital Episode Statistics |
| **Medical indication** |  |  |
| Hypertensive disorders of pregnancy | ICD10 codes I10-I15, O10-O16 | Hospital Episode Statistics |
| Diabetes | ICD10 codes E10-E14, O24 | Hospital Episode Statistics |
|  |  |  |

| **NEW SOUTH WALES** |  |  |
| --- | --- | --- |
| **Description** | **Variable** | **Data source** |
| **Caesarean section** |  |  |
| Emergency CS prelabour | Not possible to determine | N/A |
| Emergency CS in labour | Onset of labour (spontaneous, induction) + deliv98/ deliv2011= caesarean section | Perinatal Data Collection |
| Elective CS prelabour | Onset of labour (planned CS) + deliv98/ deliv2011= caesarean section | Perinatal Data Collection |
| Elective CS in labour | Not possible to determine | N/A |
| **Onset of labour** |  |  |
| Spontaneous, Induced, Planned CS | Onset of labour | Perinatal Data Collection |
| **Medical indication for type of delivery** |  |  |
| Hypertensive disorders of pregnancy (gestational hypertension, preeclampsia, eclampsia) recorded in either data source | If following variables contained any of the ICD10-AM codes: O10-O16: | Admitted Patient Data Collection |
|  | Chronic hypertension= 1, Preeclampsia=1, | Perinatal Data Collection |
|  | Gestational hypertension (with or without preeclampsia) = 1 |  |
| Diabetes mellitus in pregnancy (gestational or pre-existing) recorded in either data source | If following variables contained any of the ICD10-AM codes E10-E14, O24: | Admitted Patient Data Collection |
|  | Diabetes mellitus= 1 | Perinatal Data Collection |
|  | Gestational diabetes= 1 |  |
|  |  |  |
| **WESTERN AUSTRALIA** |  |  |
| **Description** | **Variable** | **Data source** |
| **Caesearean section** |  |  |
| Emergency | Method of birth = 8 | Midwives Notification System |
| Elective | Method of birth = 7 | Midwives Notification System |
| **Onset of labour** |  |  |
| Yes | onset of labour = 1 "spontaneous" OR = 2 "induced" | Midwives Notification System |
| No | onset of labour = 3 "no labour" |  |
| **Medical indication** |  |  |
| Hypertensive disorders of pregnancy | Based on ICD-10-AM codes: |  |
|  | Complications of Pregnancy = "pre-eclampsia" OR "gestational hypertension" OR "pre-eclampsia superimposed on essential hypertension" | Midwives Notification System |
|  | Medical Conditions = "essential hypertension" |  |
| Diabetes | Complications of Pregnancy = "gestational diabetes" | Midwives Notification System |
|  | Medical Conditions = "type 1 diabetes" OR "type 2 diabetes" |  |
