# Supplementary material for: Mode of birth and risk of infection-related hospitalisation in childhood: A population cohort study of 7.17 million births from 4 high-income countries
Source: PLoS Med. 2020 Nov 19;17(11):e1003429. doi: 10.1371/journal.pmed.1003429 (PMC7676705; doi:10.1371/journal.pmed.1003429)
Supplement: S2 Table — Estimates not available for New South Wales (Australia), Scotland, and England. Estimates are from recurrent events models fitted for total time. Models adjusted for sex, gestational age, birth weight z-score, smoking during pregnancy, maternal age at birth, parity, area level deprivation, birth year, medical indication for type of delivery, and season of birth. (DOCX) [file pmed.1003429.s007.docx]

**S2 Table: Risk of infection-related hospitalisation by mode of birth and presence of labour**

|  | **Western Australia** | **Denmark** |
| --- | --- | --- |
| **Mode of Birth** | **Hazard Ratio (95% CI)** | **Hazard Ratio (95% CI)** |
| Vaginal birth | ref | ref |
| All Emergency Caesarean Sections | 1.11 (1.09-1.13) | 1.12 (1.10-1.13) |
| Emergency Caesarean Section no labour | 1.10 (1.06-1.14) | 1.13 (1.11-1.16) |
| Emergency Caesarean Section with labour | 1.11 (1.09-1.13) | 1.11 (1.09-1.13) |
| All Elective Caesarean Sections | 1.13 (1.11-1.15) | 1.13 (1.11-1.14) |
| Elective Caesarean Section no labour | 1.13 (1.11-1.15) | 1.13 (1.11-1.15) |
| Elective Caesarean Section with labour | N/A | 1.11 (1.05-1.14) |

Estimates not available for New South Wales (Australia), Scotland, and England. Estimates are from recurrent events models fitted for total time. Models adjusted for: sex, gestational age, birth weight z-score, smoking during pregnancy, maternal age at birth, parity, area level deprivation, birth year, medical indication for type of delivery, and season of birth.
