# Supplementary material for: Mode of birth and risk of infection-related hospitalisation in childhood: A population cohort study of 7.17 million births from 4 high-income countries
Source: PLoS Med. 2020 Nov 19;17(11):e1003429. doi: 10.1371/journal.pmed.1003429 (PMC7676705; doi:10.1371/journal.pmed.1003429)
Supplement: S3 Table — Estimates are from recurrent events models fitted for total time. Fully adjusted model adjusts for sex, gestational age, birth weight z-score, maternal age at birth, parity (except for models that specify that parity was not adjusted for), area level deprivation, birth year, medical indication for type of delivery, and season of birth. (DOCX) [file pmed.1003429.s008.docx]

**S3 Table: Sensitivity analysis -parity adjustments and restricted birth years, English data**

| **English data** |  |  |  |  |  |  |
| --- | --- | --- | --- | --- | --- | --- |
|  | **All births (Apr 1998-Mar 2012)** | | | | **Births restricting  (Apr 2003 - Mar 2012)** | |
|  |  | **Fully adjusted** | **Model not adjusted for parity** | |  | **Fully adjusted** |
|  |  | **Total population** | **Total population** | **Population with known parity status** |  |  |
| **Mode of Birth** | **Total N** | **Hazard Ratio (95% CI)** | **Hazard Ratio  (95% CI)** | **Hazard Ratio  (95% CI)** | **Total N** | **Hazard Ratio  (95% CI)** |
| Vaginal | 3256920 | ref | ref | ref | 2301134 | ref |
| Any caesarean section | 946280 | 1.08 (1.08-1.09) | 1.08 (1.07-1.08) | 1.08 (1.07-1.08) | 693436 | 1.08 (1.08-1.09) |
| Emergency caesarean section | 577274 | 1.05 (1.05-1.06) | 1.04 (1.04-1.05) | 1.04 (1.04-1.05) | 425036 | 1.05 (1.04-1.06) |
| Elective caesarean section | 369006 | 1.13 (1.13-1.14) | 1.14 (1.13-1.15) | 1.14 (1.13-1.15) | 268400 | 1.13 (1.12-1.14) |

Estimates are from recurrent events models fitted for total time. Fully adjusted model adjusts for: sex, gestational age, birth weight z-score, maternal age at birth, parity (except for models that specify that parity was not adjusted for), area level deprivation, birth year, medical indication for type of delivery, and season of birth.

Annual number of matched mother-infant pairs in Maternity HES (MHES) referenced to the annual number of births in England as collected by the Office for National Statistics (ONS)

| **Financial year** | **ONS registered births in England** | **MHES linked mother-infant pairs** | **Ratio of linked MHES pairs to ONS registrations (%)** |
| --- | --- | --- | --- |
| 1998 | 598,950 | 378,879 | 63.3 |
| 1999 | 585,308 | 398,102 | 68.0 |
| 2000 | 570,556 | 394,355 | 69.1 |
| 2001 | 564,235 | 418,552 | 74.2 |
| 2002 | 571,745 | 475,793 | 83.2 |
| 2003 | 594,184 | 528,884 | 89.0 |
| 2004 | 608,645 | 540,710 | 88.8 |
| 2005 | 618,708 | 553,967 | 89.5 |
| 2006 | 640,650 | 571,723 | 89.2 |
| 2007 | 663,781 | 577,450 | 87.0 |
| 2008 | 667,932 | 598,686 | 89.6 |
| 2009 | 674,949 | 622,717 | 92.3 |
| 2010 | 682,892 | 631,106 | 92.4 |
| 2011 | 689,582 | 644,294 | 93.4 |
| Total | 8,737,052 | 7,335,218 | 84.0 |
