# Supplementary material for: Mode of birth and risk of infection-related hospitalisation in childhood: A population cohort study of 7.17 million births from 4 high-income countries
Source: PLoS Med. 2020 Nov 19;17(11):e1003429. doi: 10.1371/journal.pmed.1003429 (PMC7676705; doi:10.1371/journal.pmed.1003429)
Supplement: S4 Table — Estimates are from first event models adjusted for: sex, gestational age, birth weight z-score, smoking during pregnancy, maternal age at birth, parity, area level deprivation, birth year, medical indication for type of delivery, and season of birth. (DOCX) [file pmed.1003429.s009.docx]

**S4 Table: Sensitivity analysis - prenatal antibiotic use and gestational age, Danish data**

|  | **All births** | **All births** | **Births ≥ 30 weeks** |
| --- | --- | --- | --- |
|  | **Fully adjusted** | **Fully adjusted  + prenatal antibiotic use** | **Fully adjusted** |
| **Mode of Birth** | **Hazard Ratio (95% CI)** | **Hazard Ratio (95% CI)** | **Hazard Ratio (95% CI)** |
| **Vaginal** | ref | ref | ref |
| **Any caesarean section** | 1.13 (1.11-1.14) | 1.12 (1.11-1.14) | 1.13(1.11-1.14) |
| **Emergency caesarean section** | 1.13 (1.11-1.15) | 1.12 (1.11-1.14) | 1.13 (1.11-1.14) |
| **Elective caesarean section** | 1.13 (1.11-1.15) | 1.12 (1.11-1.14) | 1.13 (1.11-1.15) |

Estimates are from 1st event models adjusted for: sex, gestational age, birth weight z-score, smoking during pregnancy, maternal age at birth, parity, area level deprivation, birth year, medical indication for type of delivery, and season of birth.
