# Supplementary material for: Mode of birth and risk of infection-related hospitalisation in childhood: A population cohort study of 7.17 million births from 4 high-income countries
Source: PLoS Med. 2020 Nov 19;17(11):e1003429. doi: 10.1371/journal.pmed.1003429 (PMC7676705; doi:10.1371/journal.pmed.1003429)
Supplement: S5 Table — *All other infection-related hospitalisation excluded from the analyses. Asthma was identified with ICD-10 code J45; wheeze was identified with ICD-10 code R06.2. Estimates are from recurrent events models fitted for total time. Models adjusted for: sex, gestational age, birth weight z-score, smoking during pregnancy, maternal age at birth, parity, area level deprivation, birth year, medical indication for type of delivery, and season of birth. (DOCX) [file pmed.1003429.s010.docx]

**S5 Table: Sensitivity analysis – Hazard ratios for infection-related hospitalisations with and without a concurrent diagnosis of asthma and/or wheeze, Western Australia data**

|  | **Western Australia specific estimates** | | |
| --- | --- | --- | --- |
|  | **Infection-related hospitalisations with asthma/wheeze*** | **Infection-related hospitalisations without asthma/wheeze*** | **All infection-related hospitalisations** |
| **Mode of birth** | **Adjusted HR (95% CI)** | **Adjusted HR (95% CI)** | **Adjusted HR (95% CI)** |
| Vaginal | ref | ref | ref |
| Any caesarean section | 1.15 (1.10-1.21) | 1.12 (1.10-1.14) | 1.12 (1.10-1.13) |
| Emergency caesarean section | 1.17 (1.10-1.24) | 1.11 (1.08-1.13) | 1.11 (1.09-1.13) |
| Elective caesarean section | 1.14 (1.08-1.21) | 1.14 (1.12-1.16) | 1.13 (1.11-1.15) |

*All other infection-related hospitalisation excluded from the analyses.

Asthma was identified with ICD-10 code J45; wheeze was identified with ICD-10 code R06.2.

Estimates are from recurrent events models fitted for total time. Models adjusted for: sex, gestational age, birth weight z-score, smoking during pregnancy, maternal age at birth, parity, area level deprivation, birth year, medical indication for type of delivery, and season of birth.
