# Supplementary material for: Mode of birth and risk of infection-related hospitalisation in childhood: A population cohort study of 7.17 million births from 4 high-income countries
Source: PLoS Med. 2020 Nov 19;17(11):e1003429. doi: 10.1371/journal.pmed.1003429 (PMC7676705; doi:10.1371/journal.pmed.1003429)
Supplement: S6 Table — Estimates are from first event models adjusted for: sex, gestational age, birth weight z-score, smoking during pregnancy, maternal age at birth, parity, area level deprivation, birth year (overall estimate only), medical indication for type of delivery, and season of birth. (DOCX) [file pmed.1003429.s011.docx]

**S6 Table: Sensitivity analysis– Risk of infection-related hospitalisation by birth year, Western Australia data**

|  |  | **Year of birth** | | | |
| --- | --- | --- | --- | --- | --- |
|  | **Overall** | **1996-1999** | **2000-2003** | **2004-2007** | **2008-2012** |
| **Mode of birth** | HR (95% CI) | HR (95% CI) | HR (95% CI) | HR (95% CI) | HR (95% CI) |
| Vaginal | ref | ref | ref | ref | ref |
| Any caesarean section | 1.13 (1.11-1.14) | 1.15 (1.10-1.19) | 1.13 (1.10-1.16) | 1.11 (1.07-1.14) | 1.13 (1.10-1.16) |

Estimates are from 1st event models adjusted for: sex, gestational age, birth weight z-score, smoking during pregnancy, maternal age at birth, parity, area level deprivation, birth year (overall estimate only), medical indication for type of delivery, and season of birth.
