# Supplementary material for: Mode of birth and risk of infection-related hospitalisation in childhood: A population cohort study of 7.17 million births from 4 high-income countries
Source: PLoS Med. 2020 Nov 19;17(11):e1003429. doi: 10.1371/journal.pmed.1003429 (PMC7676705; doi:10.1371/journal.pmed.1003429)
Supplement: S7 Table — Calculated E-values for hazard ratios with outcome prevalence >15%. The E-value is defined as the minimum strength of association, on the risk ratio scale, that an unmeasured confounder would need to have with both the treatment and the outcome to fully explain away a specific treatment–outcome association, conditional on the measured covariates. VanderWeele TJ, Ding P. Sensitivity Analysis in Observational Research: Introducing the E-Value. Ann Intern Med. 2017;167(4):268–74. (DOCX) [file pmed.1003429.s012.docx]

**S7 Table: Sensitivity analysis – E-values**

|  | **Emergency caesarean section** | | **Elective caesarean section** | |
| --- | --- | --- | --- | --- |
|  | **Estimated HR (95% CI)** | **E-value for point estimate and lower limit of confidence interval** | **Estimated HR (95% CI)** | **E-value for point estimate and lower limit of confidence interval** |
| **Denmark** | 1.12 (1.10-1.13) | 1.38, 1.34 | 1.13 (1.11-1.14) | 1.40, 1.36 |
| **Scotland** | 1.08 (1.06-1.10) | 1.30, 1.25 | 1.11 (1.09-1.13) | 1.36, 1.32 |
| **England** | 1.05 (1.04-1.06) | 1.22, 1.20 | 1.13 (1.13-1.14) | 1.40, 1.40 |
| **New South Wales** | 1.09 (1.07-1.10) | 1.32, 1.27 | 1.12 (1.11-1.13) | 1.38, 1.36 |
| **Western Australia** | 1.11 (1.09-1.13) | 1.36, 1.32 | 1.13 (1.11-1.15) | 1.40, 1.36 |
|  |  |  |  |  |

Calculated E-values for hazard ratios with outcome prevalence >15%.

The E-value is defined as the minimum strength of association, on the risk ratio scale, that an unmeasured confounder would need to have with both the treatment and the outcome to fully explain away a specific treatment-outcome association, conditional on the measured covariates.

VanderWeele TJ, Ding P. Sensitivity Analysis in Observational Research: Introducing the E-Value. Ann Intern Med. 2017;167(4):268-74.
