# Supplementary material for: Mode of birth and risk of infection-related hospitalisation in childhood: A population cohort study of 7.17 million births from 4 high-income countries
Source: PLoS Med. 2020 Nov 19;17(11):e1003429. doi: 10.1371/journal.pmed.1003429 (PMC7676705; doi:10.1371/journal.pmed.1003429)
Supplement: S8 Table — Estimates are from first event models adjusted for: sex, gestational age, birth weight z-score, smoking during pregnancy (unless specified), maternal age at birth, parity, area level deprivation, birth year, medical indication for type of delivery, and season of birth. (DOCX) [file pmed.1003429.s013.docx]

**S8 Table: Sensitivity analysis – Hazard ratios for trauma hospitalisations, Western Australia data**

|  |  |  | **Unadjusted** | **Fully adjusted** | **Model not adjusted for smoking** |
| --- | --- | --- | --- | --- | --- |
| **Mode of Birth** | **Total N** | **Cases** | **Hazard Ratio (95% CI)** | **Hazard Ratio (95% CI)** | **Hazard Ratio (95% CI)** |
| **Vaginal** | 308575 | 12014 | ref | ref | ref |
| **Any caesarean section** | 128367 | 4678 | 0.97 (0.94-1.01) | 1.02 (0.98-1.06) | 1.02 (0.99-1.06) |
| **Emergency caesarean section** | 57933 | 2029 | 0.95 (0.91-1.00) | 1.01 (0.95-1.06) | 1.00 (0.95-1.05) |
| **Elective caesarean section** | 70434 | 2649 | 0.99 (0.95-1.03) | 1.03 (0.99-1.08) | 1.04 (1.00-1.09) |

Estimates are from 1st event models adjusted for: sex, gestational age, birth weight z-score, smoking during pregnancy (unless specified), maternal age at birth, parity, area level deprivation, birth year, medical indication for type of delivery, and season of birth.
