# Supplementary material for: Mode of birth and risk of infection-related hospitalisation in childhood: A population cohort study of 7.17 million births from 4 high-income countries
Source: PLoS Med. 2020 Nov 19;17(11):e1003429. doi: 10.1371/journal.pmed.1003429 (PMC7676705; doi:10.1371/journal.pmed.1003429)
Supplement: S9 Table — Population attributable fractions are defined as the proportion of all cases (i.e., children admitted to hospital with an infection) in the population that could be attributed to the exposure (i.e., cesarean section). *Subpopulation of emergency cesarean section and vaginal births and cases only. Elective cesarean section births and cases are excluded. **Subpopulation of elective cesarean section and vaginal births and cases only. Emergency cesarean section births and cases are excluded. Mansournia Mohammad Ali, Altman Douglas G. Population attributable fraction. BMJ. 2018;360:k757. (DOCX) [file pmed.1003429.s014.docx]

**S9 Table: Population attributable fractions**

|  | Adjusted HR | | | Prevalence of exposure among cases Pc | | | Population Attributable Fraction Pc(1-1/HR) | | | Number of children with infections attributable to caesarean section | | |
| --- | --- | --- | --- | --- | --- | --- | --- | --- | --- | --- | --- | --- |
|  | Caesarean Section | | | Caesarean Section | | | Caesarean Section | | | Caesarean Section | | |
|  | Any | Emergency | Elective | Any | Emergency* | Elective** | Any | Emergency* | Elective** | Any | Emergency* | Elective** |
| Denmark | 1.12 | 1.12 | 1.13 | 0.19 | 0.12 | 0.09 | 2.0% | 1.3% | 1.1% | 4030 | 2302 | 1855 |
| Scotland | 1.09 | 1.08 | 1.11 | 0.27 | 0.18 | 0.13 | 2.2% | 1.3% | 1.3% | 3240 | 1693 | 1624 |
| England | 1.08 | 1.05 | 1.13 | 0.25 | 0.17 | 0.11 | 1.8% | 0.8% | 1.3% | 14856 | 6006 | 8564 |
| New South Wales | 1.11 | 1.09 | 1.12 | 0.29 | 0.15 | 0.19 | 2.9% | 1.2% | 2.0% | 6668 | 2341 | 4171 |
| Western Australia | 1.12 | 1.11 | 1.13 | 0.29 | 0.16 | 0.18 | 3.2% | 1.6% | 2.1% | 3876 | 1649 | 2247 |
| Total |  |  |  |  |  |  |  |  |  | 32670 | 13991 | 18462 |

Population attributable fractions are defined as the proportion of all cases (ie. children admitted to hospital with an infection) in the population that could be attributed to the exposure (ie. caesarean section).

*Subpopulation of emergency caesarean section and vaginal births and cases only. Elective caesarean section births and cases are excluded.

**Subpopulation of elective caesarean section and vaginal births and cases only. Emergency caesarean section births and cases are excluded.

Mansournia Mohammad Ali, Altman Douglas G. Population attributable fraction BMJ 2018; 360 :k757
