# Supplementary material for: Mode of birth and risk of infection-related hospitalisation in childhood: A population cohort study of 7.17 million births from 4 high-income countries
Source: PLoS Med. 2020 Nov 19;17(11):e1003429. doi: 10.1371/journal.pmed.1003429 (PMC7676705; doi:10.1371/journal.pmed.1003429)
Supplement: Attachment — Submitted filename: PLOS Med response to reviewers.docx [file pmed.1003429.s015.docx]

17 August, 2020

PMEDICINE-D-20-00075R1

Dear PLOS Medicine editors and reviewers,

Thank you very much for considering our manuscript and for taking the time to provide very informed and detailed comments. We have addressed each comment carefully, and when needed, have performed additional analyses. Specifically, we have further addressed issues of potential bias and unmeasured confounding. We have substantially revised the manuscript including rewriting the discussion, as suggested, and feel that is has improved as a result. Thank you for considering our revised manuscript.

Sincerely,

Jessica Miller

**Requests from the editors:**
1- Please adjust the Title slightly: “Mode Of Birth And Risk Of Infection-Related Hospitalisation In Childhood: A Population Cohort Study Of 7.17 Million Births From Four Countries”

RESPONSE: We have adjusted the title as suggested.

2- Thank you for acknowledging the limits on sharing the data used in this study. Please update your data statement to include contact information (email or website) where readers may request access to each of the datasets used here.

RESPONSE: We have updated the data statement in the submission form to the following:

Regulations regarding access to jurisdictional data vary. Please visit the following websites or contact the site-specific investigators:

Denmark: contact Lars Henning Pedersen, [lhp@clin.au.dk](mailto:lhp@clin.au.dk)

England: Information on mortality and hospital episode statistics can be obtained from NHS Digital: [www.digital.nhs.uk](http://www.digital.nhs.uk)

Scotland: Information can be found at: [https://publichealthscotland.scot/](https://protect-au.mimecast.com/s/y7ZpCq717ycOmYxkHZGqlZ?domain=publichealthscotland.scot/) or contact [phs.edris@nhs.net](mailto:phs.edris@nhs.net)

New South Wales, Australia: Information regarding access to similar data used in this project can be found through NSW Centre for Health Record Linkage: [https://www.cherel.org.au/](https://protect-au.mimecast.com/s/XuClCnx1xvhGMWPLH9IIMQ?domain=cherel.org.au/)

Western Australia: Information regarding data access can be found through the Data Linkage Western Australia website: <https://www.datalinkage-wa.org.au/>

3- Please ensure that the study is reported according to the STROBE or RECORD guideline, and include the completed checklist as Supporting Information.

Please add the following statement, or similar, to the Methods: "This study is reported as per the Strengthening the Reporting of Observational Studies in Epidemiology (STROBE) guideline (S_ Checklist)."

The STROBE guideline can be found here: [http://www.equator-network.org/reporting-guidelines/strobe/](https://protect-au.mimecast.com/s/v9rwCxngnJcJRy4yu9ddlx?domain=equator-network.org)

The RECORD guideline can be found here: [https://www.equator-network.org/reporting-guidelines/record/](https://protect-au.mimecast.com/s/pZifCyojoKsN2gzgIBAOBA?domain=equator-network.org)

When completing the checklist, please use section and paragraph numbers, rather than page numbers.

RESPONSE: The RECORD checklist has been revised to include section and paragraph numbers from the clean revised manuscript. The below RECORD reporting statement has been added to the Methods section.

PAGE 10, LINES 278-279: *This study is reported as per the* ***RE****porting of studies* ***C****onducted using* ***O****bservational* ***R****outinely-collected* ***D****ata (****RECORD****) guideline (S1 Checklist).*

4- Did your study have a prospective protocol or analysis plan? Please state this (either way) early in the Methods section.
a) If a prospective analysis plan (from your funding proposal, IRB or other ethics committee submission, study protocol, or other planning document written before analyzing the data) was used in designing the study, please include the relevant prospectively written document with your revised manuscript as a Supporting Information file to be published alongside your study, and cite it in the Methods section. A legend for this file should be included at the end of your manuscript.
b) If no such document exists, please make sure that the Methods section transparently describes when analyses were planned, and when/why any data-driven changes to analyses took place.
c) In either case, changes in the analysis-- including those made in response to peer review comments-- should be identified as such in the Methods section of the paper, with rationale.

RESPONSE: The sites followed a standardised analysis plan, which was agreed *a priori* by the investigators. The text shown was already included in the methods. We have added the analysis plan to the supporting information and included a legend at the end of the manuscript.

PAGE 7, LINES 210-212: *Each site followed a standardised protocol for data coding and analysis to generate site-specific risk estimates for each study population, modelling risk of infection-related hospitalisation over time by mode of birth (S1 Analysis Plan).*

PAGE 18, LINE 469: *S1 Analysis Plan – Analysis plan for study populations*

5- Please structure your abstract using the PLOS Medicine headings (Background, Methods and Findings, Conclusions).

RESPONSE: We have changed the abstract headings as suggested.

6- In the abstract and throughout the manuscript, is it more accurate to describe the cohort as “all *recorded* singleton live births” or similar?

RESPONSE: We have made the suggested changes at the following locations:

*Abstract:*

PAGE 2, LINE 57: *We conducted a multi-country population-based cohort study of all recorded singleton live births…*

PAGE 2, LINE 65: *7,174,787 live recorded births were included…*

*Methods:*

PAGE 6, LINE 179: *All recorded live-born singletons…*

7- In the Abstract Methods and Findings section, please provide the full date range (Jan 1 1996– Dec 31 2015?), and clarify that not all cohorts covered this entire range.

RESPONSE: We have added the full date range at the following locations:

Abstract

PAGE 2, LINES 56-60: *We conducted a multi-country population-based cohort study of all recorded singleton live births from January 1 1996–December 31 2015, using record-linked birth and hospitalisation data from Denmark, Scotland, England, and Australia (New South Wales and Western Australia). Birth years within the date range varied by site but data were available from at least 2001-2010 for each site.*

Methods

PAGE 6, LINES 180-181: *Birth years ranged from January 1 1996-December 31 2015 with data from at least 2001-2010 available from each site.*

8- In the Abstract and throughout the manuscript, please include p-values alongside the 95% CIs.

RESPONSE: We have included the p-values in the abstract and throughout the manuscript at the following locations:

Abstract

PAGE 2, LINES 67-71: *Risk was greater among CS-born versus vaginally-born children (hazard ratio from random effects model, HR 1.10, 95% CI 1.09-1.12, p<0.001, and slightly higher following elective than emergency CS (HR 1.13, 95% CI 1.12-1.13, p<0.001 versus HR 1.09, 95% CI 1.06-1.12, p<0.001).*

Results

PAGE 11, LINES 305-308: *CS-born children had an increased risk of an infection-related hospitalisation compared with vaginally-born children (hazard ratio from random effects model, HR 1.10, 95% CI 1.09-1.12, p<0.001). Elective CS had a slightly stronger association (HR 1.13, 95% CI 1.12-1.13, p<0.001) than emergency CS (HR 1.09, 95% CI 1.06-1.12, p<0.001)…*

PAGE 11, LINES 313-3143: *A higher relative risk was observed when labour did not occur compared with births following labour (HR 1.12, 95% CI 1.12-1.13, p<0.001)…*

PAGE 11, LINES 318-319: *… CS was similar to overall findings (elective CS HR 1.14, 95% CI 1.12-1.15, p<0.001; emergency CS HR 1.08, 95% CI 1.04-1.12, p<0.001)…*

9- In the last sentence of the Abstract Methods and Findings section, please describe the main limitation(s) of the study's methodology.

RESPONSE: The main limitations have been added to the Abstract at the following location:

PAGE 2, LINES 74-75: *Limitations include site-specific and longitudinal variations in clinical practice and in the definition and availability of some data. Data on postnatal factors were not* *available.*

10- In the Abstract Conclusions, please address the study implications without overreaching what can be concluded from the data; the phrase "In this study, we observed ..." may be useful.

RESPONSE: We have changed the Abstract Conclusions to the following:

PAGE 2, LINES 78-82: *In this study, we observed a consistent association between birth by CS and infection-related hospitalisation in early childhood. Notwithstanding the limitations of observational data, the associations may reflect differences in early microbial exposure by mode of birth, which should be investigated by mechanistic studies. If our findings are confirmed, they could inform efforts to reduce elective CS rates that are not clinically indicated.*

11- At this stage, we ask that you include a short, non-technical Author Summary of your research to make findings accessible to a wide audience that includes both scientists and non-scientists. The Author Summary should immediately follow the Abstract in your revised manuscript. This text is subject to editorial change and should be distinct from the scientific abstract. Please see our author guidelines for more information: [https://journals.plos.org/plosmedicine/s/revising-your-manuscript#loc-author-summary](https://protect-au.mimecast.com/s/iR-JCzvkvLcRw3p3cO9Ibj?domain=journals.plos.org)

RESPONSE: The below Author Summary has been added to the text:

**Why Was This Study Done?**

- Health outcomes beyond the neonatal period for children born by caesarean section are not well understood.
- Caesarean section may be associated with an increased risk of severe childhood infection requiring hospitalisation, but data are limited.
- Whether caesarean section is associated with increased risk of overall infection or only certain types of infection and whether the risk differs for emergency versus elective caesarean section is unclear.

**What Did the Researchers Do and Find?**

- **Using total population birth and hospitalisation data** from Denmark, Scotland, England, and Australia (New South Wales and Western Australia**), we followed all recorded singleton live births from January 1 1996-December 31 2015 for up to 5 years to determine whether children were admitted to hospital with an infection**.
- We estimated risk of overall and clinical type of infection by mode of birth, vaginal or caesarean section (emergency/elective).
- Among 7.17 million births, children born by elective caesarean section, compared to vaginally-born children, had a 13% increased risk for an infection-related hospitalisation and emergency caesarean section-born children had a 9% increased risk.
- Increased risks persisted to 5 years of age and were highest for respiratory, gastrointestinal, and other viral infections.

**What Do These Findings Mean?**

- In our large multinational study, we observed a consistent association between birth by caesarean section and infection-related hospitalisation in early childhood.
- Limitations include site-specific and longitudinal variations in clinical practice.
- The associations may reflect differences in early microbial exposure by mode of birth, which should be investigated by mechanistic studies.
- These findings may contribute to the global effort to reduce the rates of elective caesarean section that are not medically indicated.

12- In the Results section, please include the unadjusted HRs along with the adjusted HRs.

RESPONSE: In the interests of clarity and brevity, we did not include the unadjusted HRs in the Results section. However, in Figure 1 of our overall estimates, we include the site-specific unadjusted HRs. We did not perform a meta-analysis of the unadjusted HRs, as there are a number of covariates that are well-recognised to be associated with either mode of birth and/or infection-related hospitalisation and we therefore felt that unadjusted results would be difficult to interpret in meta-analysis. We therefore only performed a meta-analysis of the adjusted HRs.

13- Please re-organize the Discussion and present as follows: a short, clear summary of the article's findings; what the study adds to existing research and where and why the results may differ from previous research; strengths and limitations of the study; implications and next steps for research, clinical practice, and/or public policy; one-paragraph conclusion.

RESPONSE: We have extensively re-organized and shortened the Discussion as suggested (PAGES 12-16, LINES 346-448).

14- In the Conclusion, please be a bit more specific regarding the implications and recommendations for future research.

RESPONSE: We have rewritten the Conclusion as suggested:

PAGE 16, LINES 446-448: *In conclusion, in a large multinational study, children born by CS were at increased risk of infection-related hospitalisation until age five years. These findings may contribute to the global effort to reduce the rates of CS that are not medically indicated.*

15- Please place reference numbers in square brackets preceding punctuation. Please also make sure to use the "Vancouver" style for reference formatting, and see our website for other reference guidelines [https://journals.plos.org/plosmedicine/s/submission-guidelines#loc-references](https://protect-au.mimecast.com/s/uBS9CANpN0h9lg3gTpsAUq?domain=journals.plos.org)

RESPONSE: We have edited the reference placement and formatting as suggested.

16- Please provide titles and legends for each individual table and figure in the Supporting Information and list all SI files at the end of the manuscript text.

RESPONSE: We have added all titles and legends for each table and figure in the Supporting Information at the end of the manuscript text as follows (PAGES 18-19, LINES 468-535):

**Supporting Information**

**S1 Checklist – RECORD checklist**

**S1 Analysis Plan – Analysis plan for study populations**

**S1 Figure - Flow charts for site-specific study populations**

**S1 Table – Variable definition**

**S2 Figure: Site-specific and meta-analysis hazard ratios for infection-related hospitalisation in births without labour**

Estimates are from recurrent events models fitted for total time. Models adjusted for: sex, gestational age, birth weight z-score, smoking during pregnancy, maternal age at birth, parity, area level deprivation, birth year, medical indication for type of delivery, and season of birth. Reference is births with labour. I-V: inverse-variance weighted fixed effects model, D+L: DerSimonian and Laird random effects model.

**S2 Table: Risk of infection-related hospitalisation by mode of birth and presence of labour**

Estimates not available for New South Wales (Australia), Scotland, and England. Estimates are from recurrent events models fitted for total time. Models adjusted for: sex, gestational age, birth weight z-score, smoking during pregnancy, maternal age at birth, parity, area level deprivation, birth year, medical indication for type of delivery, and season of birth.

**S3 Table: Sensitivity analysis -parity adjustments and restricted birth years, English data**

Estimates are from recurrent events models fitted for total time. Fully adjusted model adjusts for: sex, gestational age, birth weight z-score, maternal age at birth, parity (except for models that specify that parity was not adjusted for), area level deprivation, birth year, medical indication for type of delivery, and season of birth.

**S4 Table: Sensitivity analysis - prenatal antibiotic use and gestational age, Danish data**

Estimates are from 1st event models adjusted for: sex, gestational age, birth weight z-score, smoking during pregnancy, maternal age at birth, parity, area level deprivation, birth year, medical indication for type of delivery, and season of birth.

**S3 Figure: Site-specific and meta-analysis hazard ratios for infection-related hospitalisation, not adjusted for smoking during pregnancy**

Estimates are from recurrent events models fitted for total time. Models adjusted for: sex, gestational age, birth weight z-score, maternal age at birth, parity, area level deprivation, birth year, medical indication for type of delivery, and season of birth. Reference is vaginal births. I-V: inverse-variance weighted fixed effects model, D+L: DerSimonian and Laird random effects model.

**S5 Table: Sensitivity analysis – Hazard ratios for infection-related hospitalisations with and without a concurrent diagnosis of asthma and/or wheeze, Western Australia data**

*All other infection-related hospitalisation excluded from the analyses.

Asthma was identified with ICD-10 code J45; wheeze was identified with ICD-10 code R06.2.

Estimates are from recurrent events models fitted for total time. Models adjusted for: sex, gestational age, birth weight z-score, smoking during pregnancy, maternal age at birth, parity, area level deprivation, birth year, medical indication for type of delivery, and season of birth.

**S6 Table: Sensitivity analysis – E-values**

Calculated E-values for hazard ratios with outcome prevalence >15%.

The E-value is defined as the minimum strength of association, on the risk ratio scale, that an unmeasured confounder would need to have with both the treatment and the outcome to fully explain away a specific treatment-outcome association, conditional on the measured covariates.

VanderWeele TJ, Ding P. Sensitivity Analysis in Observational Research: Introducing the E-Value. Ann Intern Med. 2017;167(4):268-74.

**S7 Table: Sensitivity analysis – Hazard ratios for trauma hospitalisations, Western Australia data**

Estimates are from 1st event models adjusted for: sex, gestational age, birth weight z-score, smoking during pregnancy (unless specified), maternal age at birth, parity, area level deprivation, birth year, medical indication for type of delivery, and season of birth.

**S8 Table: Population attributable fractions**

Population attributable fractions are defined as the proportion of all cases (ie. children admitted to hospital with an infection) in the population that could be attributed to the exposure (ie. caesarean section).

*Subpopulation of emergency caesarean section and vaginal births and cases only. Elective caesarean section births and cases are excluded.

**Subpopulation of elective caesarean section and vaginal births and cases only. Emergency caesarean section births and cases are excluded.

Mansournia Mohammad Ali, Altman Douglas G. Population attributable fraction *BMJ* 2018; 360 :k757

Comments from the reviewers:

**Reviewer #1:** Miller et al. present the findings of a multi-country analysis evaluating the association between children born by CS compared to vagina delivery and infection-related hospitalisation. The statistical methods are straightforward, using multivariate Cox regression models to determine the AHRs for harmonised across country-specific data, and pooled into an aggregate figure in a two-stage process used fixed and random-effects meta-analysis. The results are largely consistent with previous studies, but this study does have a much larger sample size, include broader range of IRHs, and use some additional methods to address residual confounding, including using DAGs to graphically identify confounders, and E-values to measure the potential effects of unmeasured confounding on the results. The results are quite consistent across all four countries, and should generalise to developed countries with similar quality of obstetrics care.

Major comments
1) I thought what would strengthen the case for this paper is the authors could elaborate a bit further in the discussion what this particular adds compared to previous Australian (ref 13) and Danish studies (ref 7). Both these previous studies are not small either (Australian study n = 212k; Danish study n = 750k) which also quite similar results (11-20% increased risk). That means highlighting what the limitations of these previous studies may have been and what this current study has improved upon. As it stands, the study feels largely like a confirmatory study of previous results (which itself is important aspect of science) but I think the authors could enhance the novelty of their approach a bit further.

RESPONSE: The previous studies cited are from Australia (Ref 13, Moore et al, 2012) and Denmark (Ref 7, Kristensen et al, 2016), and we have added another recent study (Ref 15, Wainstock et al, 2019) as suggested by Reviewer #4. All three are large, well-conducted, population-based studies and make important contributions. However, these studies have shared and unique limitations that we attempted to address in the current study. Each is from a single jurisdiction and so findings may in part reflect local population characteristics and clinical practices, which are hard to account for in single-centre analyses. Studies from single countries/centres can also be limited by issues of data quality and/or completeness, which may result in idiosyncratic biases. Our study is overwhelmingly larger (enabling highly powered subgroup analyses) and, importantly, used data from a number of different sources covering different populations, clinical practice and culture, and included countries with different CS rates. Similar associations were found across all sites, which gives confidence that the associations reported (and underlying data) are robust.

Addressing the study-specific limitations of those previous publications, **the Danish study** had a number of outcomes broadly related to ‘immune function’ including some infections, autoimmune diseases and some haematological cancers. Although mode of birth may impact on immune function broadly, it is likely that different and additional causal pathways are involved for these heterogeneous outcomes, particularly genetics and other environmental exposures that were not measured. Confounding by indication may be particularly important in this context and this was not addressed. The diagnostic outcome categories in the Danish study did not capture all the possible sub-groups of infection, auto-immune disease and malignancy, which makes interpretation more difficult. Finally, the number of cases for almost all the non-infectious outcomes were small and firm conclusions could not be drawn.
In the current study, we focused solely on infection, the commonest reason for childhood hospitalisation and an outcome with marked differential severity despite universal exposure to causative pathogens.

In **the Western Australian study** (Moore et al), the outcomes in that study were focused solely on common acute lower respiratory infections, namely bronchiolitis and pneumonia in the first 2 years of life. Models investigating the risk of elective caesarean were estimated separately for the first and second years of life. The authors adjusted for similar co-variates as we have in the current study. Confounding by indication was not addressed in the Western Australian study. Additionally, we did not limit our current analysis to term births as was done in the Moore et al study. Most importantly, our current analysis included a longer period of follow-up (5 years rather than 2 years), assessed all infection-related hospitalisation outcomes and included a more granular analysis of the postnatal age periods at which we assessed risk (0-3 months, 4-6 months, 7-12 months, 1-2 years and 2-5 years), which we considered important to assess whether any associations persist across the pre-school period, when infection is most common.

**The** **Israeli study** (Wainstock et al), had a similar outcome of all infection-related hospitalisations (first events only) and children were followed from birth to 18 years of age. The study was from a single tertiary centre and was restricted to uncomplicated pregnancies and deliveries. Only vaginal and elective caesarean section births were included. The reported hazard ratio for all elective CS births (HR: 1.18, 95%CI: 1.11-1.25) was slightly higher than in our study, one possibility due to inclusion of neonatal infections before the birth-related discharge. In our study, we considered multiple hospitalisations in the child, rather than just the first event and, as previously stated, we performed a more granular analysis of the postnatal age period.

In summary, the current study builds significantly on these prior large-scale population-based analyses and represents a unique and highly citeable contribution to the field in terms of sample size; data from multiple jurisdictions; complete ascertainment of all infection-related hospitalisations; longer and more granular period of follow-up; and a considered *a priori* approach to addressing the key issue of confounding by indication. In the interests of brevity, we have edited the following text to the Introduction to encapsulate these differences in relation to previously published analyses:

PAGE 5, LINES 156-163: *A recent Israeli study among uncomplicated pregnancies and births (n = 138,910) estimated a 10% and 23% increased risk of hospitalisations with infection up to age 18 years among term only and preterm only elective caesarean section births, respectively [15]. These studies are from single jurisdictions where population-specific characteristics and obstetric practice may have unknown effects. No studies have investigated all infection-related hospitalisations and it is unclear if CS is associated with increased risk of overall infection-related hospitalisation or only certain infection types, whether risk differs for emergency versus elective CS, and if the associations may partly represent confounding by indication.*

2) The primary limitation the authors eluded to in the introduction and discussion (which I agree with) is confounding by indication. As a randomised trial is clearly not possible, a causal relationship remains elusive. Whilst the authors have given this thought, through use of DAGS to identify potential confounders and quantify the influence through E-values, the main multivariate statistical analysis does not actually attempt to address this issue. I thought there was a clear opportunity here to use a propensity score adjustment in the model ([https://www.ahajournals.org/doi/10.1161/CIRCOUTCOMES.113.000359](https://protect-au.mimecast.com/s/oDaCCBNqNkhVR191TkuBq0?domain=ahajournals.org)) either using a matching process or just direct adjusting for the PS. The extraordinary size of the sample also creates an opportunity for covariate balancing ([https://rss.onlinelibrary.wiley.com/doi/full/10.1111/rssb.12027](https://protect-au.mimecast.com/s/ckYICD1v1msBJ1K1tQMx3f?domain=rss.onlinelibrary.wiley.com)).

This is not to say what the authors did in the analysis was incorrect (as their approach is entirely valid) but rather that their primary limitation could be partially addressed by consideration of the above statistical approaches.

RESPONSE: Propensity scores are used to balance the covariates, and potential unobserved differences, between the treatment or exposure groups. They work best when extensive covariate data are available. In our study, we decided to take the approach of a subgroup analysis among participants with similar background characteristics to balance covariates and potential unobserved differences between exposure groups. We defined our maternal sub-populations as having cephalic-presenting infants born ≥37 weeks gestational age, with birth weight between the 10-90th percentiles for gestational age and sex, maternal age of 20-34 years without any reported hypertensive disorders or diabetes preceding or during pregnancy. We believe that among this subpopulation, variation and unobserved differences were minimised. Perhaps a future study might benefit from a comparison of results between propensity score adjustment and subgroup analyses.

PAGE 13, LINES 369-371: *We analysed a pre-defined sub-population considered at low obstetric risk in whom findings were similar to the overall cohort, indicating that confounding by indication was unlikely to be responsible for the observed associations.*

3) I would have liked to seen some thought given to using a negative control outcome ([https://www.ncbi.nlm.nih.gov/pmc/articles/PMC5428075/](https://protect-au.mimecast.com/s/1LyECE8w8nsW0mJmu6jqBB?domain=ncbi.nlm.nih.gov)) to further strengthen the causal inference framework. Non-infection related hospitalisations could potentially be used here (the inverse of the outcome). The hypothesized mechanism would be that any sources of unmeasured confounding would also lead to an increase in non-infection related hospitalisation. An expected null results in the negative control outcome would remarkably strengthen the results.

RESPONSE: We agree that using a negative control is helpful for the consideration of unmeasured confounding. We have now performed and included the findings of first occurrence of trauma admissions as the negative control in the Western Australia data, where these hospital data were readily available and have been validated. Cases that were additionally diagnosed with an infection during the admission were excluded. A total of 16,692 children were admitted with a trauma diagnostic code prior to their 5^th^ birthday.

A null association was found between mode of delivery and trauma admission in the children, which gives considerable confidence to our main findings.

The following text has been added to the following sections:

Methods PAGE 9, LINES 266-270: *Similarly, we estimated the association between mode of birth and the negative control outcome of trauma in the Western Australia data, where these hospital data were readily available and have been validated. We hypothesised that if bias or unmeasured confounding is present, we might observe an increase in risk for non-infection-related trauma admissions.*

Results PAGE 12, LINES 336-338: *In the analysis of mode of birth and the negative control outcome of trauma admissions, we did not observe an association (S7 Table).*

Discussion PAGE 13, LINES 371-374: *In addition, where data were available, we analysed a negative control outcome (hospitalisation with trauma) and observed no association with mode of birth, which gives confidence that our findings are not reflective of bias or unmeasured confounding.*

**S7 Table: Sensitivity analysis – Hazard ratios for trauma hospitalisations, Western Australia data**

|  |  |  | **Unadjusted** | **Fully adjusted** | **Model not adjusted for smoking** |
| --- | --- | --- | --- | --- | --- |
| **Mode of delivery** | **Total N** | **Cases** | **Hazard Ratio (95% CI)** | **Hazard Ratio (95% CI)** | **Hazard Ratio (95% CI)** |
| **Vaginal** | 308575 | 12014 | ref | ref | ref |
| **Any caesarean section** | 128367 | 4678 | 0.97 (0.94-1.01) | 1.02 (0.98-1.06) | 1.02 (0.99-1.06) |
| **Emergency caesarean section** | 57933 | 2029 | 0.95 (0.91-1.00) | 1.01 (0.95-1.06) | 1.00 (0.95-1.05) |
| **Elective caesarean section** | 70434 | 2649 | 0.99 (0.95-1.03) | 1.03 (0.99-1.08) | 1.04 (1.00-1.09) |

Estimates are from 1st event models adjusted for: sex, gestational age, birth weight z-score, smoking during pregnancy (unless specified), maternal age at birth, parity, area level deprivation, birth year, medical indication for type of delivery, and season of birth.

Minor:


4) Abstract methods: specify whether pooled results following in the findings section of the abstract are from fixed or random-effects meta-analysis

RESPONSE: We have specified the random-effects model to the first mention of results in both the abstract and results section of the manuscript as follows:

Abstract PAGE 2, LINE 68-69: *(hazard ratio from random effects model, HR…)*

5) Methods - statistical analysis: Use of DAGS - I agree that DAGS are useful tools here for causal inference as a graphic tool but I haven't seen where the authors specifically elaborated how they were used to identify confounder in the multivariate analysis. Please elaborate on variable selection and criteria applied.

RESPONSE: The following underlined section has been added to the Methods section:

PAGE 7, LINES 216-219*: Potential confounders were identified with directed acyclic graphs, which provide a visual representation of causal assumptions. Variables were defined as potential confounders if they were associated with exposure but not affected by the exposure, i.e. not an intermediate on the causal pathway, and if they were independent risk factors for the outcome.*

6) Methods - sensitivity analysis: Was the sensitivity analysis on English data conducted with imputation of parity or was this as complete case analyses? And imputation was used - please specify the exact methodology.

RESPONSE: The sensitivity analysis for parity included running the overall model in 1) the total population with parity included in the model, 2) the total population with parity not included in the model and 3) the population restricted to those with known parity status but with parity not included in the model. Comparison of models 1 and 3 is the most fair comparison as these models cover the same population (i.e. individuals with known parity status), but dropping part of the study population in this way can sometimes introduce bias; however, imputation was not necessary here since the results from all three models were near-identical. We have now included results from all three models in the supporting information table so that all scenarios are presented, see below.

| **English data** |  |  |  |  |  |  |
| --- | --- | --- | --- | --- | --- | --- |
|  | **All births (Apr 1998-Mar 2012)** | | | | **Births restricting  (Apr 2003 - Mar 2012)** | |
|  |  | **Fully adjusted** | **Model not adjusted for parity** | |  | **Fully adjusted** |
|  |  | **Total population** | **Total population** | **Population with known parity status** |  |  |
| **Mode of delivery** | **Total N** | **Hazard Ratio (95% CI)** | **Hazard Ratio  (95% CI)** | **Hazard Ratio  (95% CI)** | **Total N** | **Hazard Ratio  (95% CI)** |
| Vaginal | 3256920 | ref | ref | ref | 2301134 | ref |
| Any caesarean section | 946280 | 1.08 (1.08-1.09) | 1.08 (1.07-1.08) | 1.08 (1.07-1.08) | 693436 | 1.08 (1.08-1.09) |
| Emergency caesarean section | 577274 | 1.05 (1.05-1.06) | 1.04 (1.04-1.05) | 1.04 (1.04-1.05) | 425036 | 1.05 (1.04-1.06) |
| Elective caesarean section | 369006 | 1.13 (1.13-1.14) | 1.14 (1.13-1.15) | 1.14 (1.13-1.15) | 268400 | 1.13 (1.12-1.14) |

Estimates are from recurrent events models fitted for total time. Fully adjusted model adjusts for: sex, gestational age, birth weight z-score, maternal age at birth, parity (except for models that specify that parity was not adjusted for), area level deprivation, birth year, medical indication for type of delivery, and season of birth.

The table illustrates that including and excluding parity from the model, and the missingness associated with parity, makes little difference to the results. Therefore, we do not believe that imputations for parity are needed. The below underlined section was added to the text:

PAGE 7, LINES 230-235: *Sensitivity analyses were conducted in the English data with and without adjusting for parity in the models, and in all sites with and without adjusting for smoking. In the English data, models that did not adjust for parity were run in both the total population and in the population restricted to those with known parity status (to help disentangle any effect of parity as a confounder from any effect of missing data).*

**Reviewer #2:** The authors analyze databases and show a consistently higher incidence of hospitalization due to infection up to 5 years of age in children born by CS versus those born by vaginal delivery in four wealthy countries.

The manuscript is clear, concise and well written.

Major comments

1. I did not find the argument convincing that changes in gut flora are likely the explanation for the differences in infection rates. My objections are:
a) Do we really know that differences in gut flora following C/S persist for years?

RESPONSE: It is unclear how long differences in the infant’s microbiome arising from perinatal factors (including mode of birth) persist. Differences are evident until at least 3-6 months postnatally with 16S PCR, which is a relatively crude measure of microbiome diversity and does not assess microbial gene expression or functional effects (PMID: 27475754). More sensitive methods can detect mode of birth-related differences in the microbiome until 36-48 months of age (<https://doi.org/10.1159/000490841> and our original ref 31). The infant microbiome is key to immune development and there is evidence that there is a critical early life window when immune responses are programmed (PMID: [31777069](https://www.ncbi.nlm.nih.gov/pubmed/31777069)). We have changed the text to the following:

PAGE 15 LINES 420-425: *Differences in initial microbial exposure by mode of birth, which may persist for months or possibly years [34], may contribute to the increased risk of infection-related hospitalisation following CS by effects on the development of postnatal immune responses. The composition and function of the early microbiome has been linked to a range of adverse short and longer term immune-mediated outcomes [9], although we are not aware of studies that have directly linked the postnatal microbiome with risk of common childhood infections.*

b) What is the direct evidence that gut flora plays any role in what infections a child gets?

RESPONSE: As exposure to infectious pathogens, especially in early childhood, is essentially universal, the immune response is the central mechanism underpinning differential susceptibility to infection. There is consensus that the early microbiome programs immune responses, so it is entirely logical that there will be a causal relationship between the early life postnatal microbiome, immune responses and susceptibility to and severity of infection. There is a rapidly increasing body of observational data to support this widely accepted premise (e.g. PMID: [30986482](https://www.ncbi.nlm.nih.gov/pubmed/30986482); PMID: 25865368, PMID: 31076501; PMID: 30883192).

c) No other theories are presented. Is it possible that women who end up with a C/S seek health care for their children more often than do women who had a vaginal delivery? The authors imply that hospitalization is an objective outcome. However there are women who do not want any intervention for themselves or for their children so are less likely to have a C/S or to have their children admitted with infection.

RESPONSE: While we acknowledge this possibility, there doesn’t appear to be empirical evidence to support this suggestion. (If we are mistaken and this suggestion can be referenced, we would be happy to include a sentence in the Discussion). Moreover, healthcare and hospital care specifically are free in the jurisdictions who provided data for this analysis, so concerns regarding direct costs are unlikely to influence the outcomes. Our considerable clinical experience is that sick children eventually end up in hospital; some may present late (which may reflect parental preference) but infection-related admissions are almost always on the basis of clinical severity, and very rarely for social reasons. In addition, the indications for CS are very heterogenous, such that it is unlikely that there will be a single stereotyped health-seeking behaviour in families of children born by CS and therefore the potential impact on the findings, if present, is likely to be small. Our results from the negative control group of trauma hospitalisations (as suggested by reviewer 1, question 3) further suggest that there is no discernible association between mode of birth and health-seeking behaviour.

2. I would make it a bit clearer that although the incidence of infection increased 10% with CS, only approximately 2% of children had an excess admission related to being born by CS. It might be helpful to tell the reader how many excess admissions this would be annually for each of the 5 jurisdictions that were studied.

RESPONSE: We agree that this could be clearer and have added the following column to the supporting information table and underlined changes in the footnote. The following changes have been made to the text:

PAGE 9, LINES 272-273: *We calculated site-specific population attributable fractions to quantify the percentage and number of infection-related hospitalisations attributable to CSs.*

PAGE 12, LINES 340-344: *Population attributable fractions are provided in S8 Table. If the associations are causal, two to three percent of children with infection-related hospitalisations could attribute their infection to being born by CS. Among the 1,502,537 children who had an infection-related hospitalisation in our study, about 14,000 calculated children had infections that could be attributed to being born by emergency CS and 18,500 to being born by elective CS.*

|  | Adjusted HR | | | Prevalence of exposure among cases Pc | | | Population Attributable Fraction Pc(1-1/HR) | | | Number of children with infections attributable to caesarean section | | |
| --- | --- | --- | --- | --- | --- | --- | --- | --- | --- | --- | --- | --- |
|  | Caesarean Section | | | Caesarean Section | | | Caesarean Section | | | Caesarean Section | | |
|  | Any | Emergency | Elective | Any | Emergency* | Elective** | Any | Emergency* | Elective** | Any | Emergency* | Elective** |
| Denmark | 1.12 | 1.12 | 1.13 | 0.19 | 0.12 | 0.09 | 2.0% | 1.3% | 1.1% | 4030 | 2302 | 1855 |
| Scotland | 1.09 | 1.08 | 1.11 | 0.27 | 0.18 | 0.13 | 2.2% | 1.3% | 1.3% | 3240 | 1693 | 1624 |
| England | 1.08 | 1.05 | 1.13 | 0.25 | 0.17 | 0.11 | 1.8% | 0.8% | 1.3% | 14856 | 6006 | 8564 |
| New South Wales | 1.11 | 1.09 | 1.12 | 0.29 | 0.15 | 0.19 | 2.9% | 1.2% | 2.0% | 6668 | 2341 | 4171 |
| Western Australia | 1.12 | 1.11 | 1.13 | 0.29 | 0.16 | 0.18 | 3.2% | 1.6% | 2.1% | 3876 | 1649 | 2247 |
| Total |  |  |  |  |  |  |  |  |  | 32670 | 13991 | 18462 |
| Population attributable fractions are defined as the proportion of all cases (ie. children admitted to hospital with an infection) in the population that could be attributed to the exposure (ie. caesarean section). | | | | | | | | | | | | |
| *Subpopulation of emergency caesarean section and vaginal births and cases only. Elective caesarean section births and cases are excluded. | | | | | | | | | | | | |
| **Subpopulation of elective caesarean section and vaginal births and cases only. Emergency caesarean section births and cases are excluded. | | | | | | | | | | | | |
|  |  |  |  |  |  |  |  |  | |  | |  |
| Mansournia Mohammad Ali, Altman Douglas G. Population attributable fraction *BMJ* 2018; 360 :k757 | | | | | | | | | | | | |

Minor comments

3. I would clarify in the abstract that infections occurring during the birth hospitalization were excluded.

RESPONSE: The following underlined text has been added to the abstract:

PAGE 2, LINES 61-63: *Infection-related hospitalisations (overall, and by clinical type) occurring after the birth-related discharge date were identified in children until five years of age by primary/secondary ICD-10 diagnosis codes.*

4. "An increased risk of specific infection-related hospitalisations, mainly lower respiratory tract and gastrointestinal infections, has been associated with CS.(11, 12)" Please provide details from the quoted studies.

RESPONSE: We did not go into the details of the findings of these studies given the word limits and because Ref 11 is a relatively small study and ref 12 is our own work and focussed on a number of factors in addition to CS that may impact the risk of gastrointestinal infection. Instead, we chose to focus on larger studies and the next sentence in the Introduction provides details of those we considered most relevant and illustrative.

5. Why were children with congenital malformations excluded? With this sample size, findings should not be affected by inclusion of children who might have a higher incidence of infections. I am not suggesting that the authors re-do the study but they ought to explain to the reader why they did this.

RESPONSE: The inclusion of those with congenital malformations was considered carefully a priori. We are mindful of the potential for confounding generally in population-level analyses and congenital anomalies were considered an avoidable, albeit relatively uncommon, confounder, as some conditions may result in increased CS rate and postnatally are associated with increased risk of infection (particularly lower respiratory tract infections). These children may also be born with severe conditions associated with increased comorbidity and make them more vulnerable and susceptible to infections. We therefore decided to remove these from our analyses, an approach we have adopted previously in similar analyses on the same reasoning (PMID: 27052469). The following text has been added:

PAGE 6, LINES 181-184: *Children with congenital malformations (International Classification of Diseases, 10th Revision (ICD10) codes Q00-Q99) were excluded as some conditions may be associated with mode of birth and postnatally with increased infection risk.*

6. Why did the authors only look at the first 3 infection related hospitalizations?

RESPONSE: We only looked at the first 3 infection-related hospitalisations because, as shown in Table 1, the proportion of children with 3 or more infection-related hospitalisations was small. The Prentice, Williams, and Peterson model that we used for recurrent events data analyses multiple events by stratification, based on the prior number of events during the follow-up period. When risk sets become very small, in our study greater than 3 infection-related hospitalisations, then the event-specific estimates become unreliable. The following was added to the methods:

PAGE 7, LINES 228-229: *Recurrent events were limited to the first 3 hospitalisations as risk sets for additional hospitalisations were very small, which may result in unreliable estimates [25].*

25.	Amorim LD, Cai J. Modelling recurrent events: a tutorial for analysis in epidemiology. Int J Epidemiol. 2015;44(1):324-33. Epub 2014/12/17. doi: 10.1093/ije/dyu222. PubMed PMID: 25501468; PubMed Central PMCID: PMCPMC4339761.

7. When did data collection end? How many children were followed for less than 5 years simply because data collection ended?

RESPONSE: Data collection ended at different times depending on the site. Table 1 provides the dates for when hospital data were available which corresponds to the study end dates for each site. Children born during the last few years of the birth range may not have been followed for a full 5 years. Our analyses of time-to-event data incorporate person-years. Table 1 illustrates that on average, children were followed from a range of 3.39-4.38 years with a standard deviation of about 1.5 years, depending on the site.

In the text we included a reference for Table 1 when we mention study end dates.

PAGE 7, LINES 212-216: *Children were followed from their birth-related hospital discharge date until an infection-related hospitalisation (up to the first three hospitalisations), death, emigration (where data were available), 5^th^ birthday, or end of study period (which varied with years of available data, Table 1), whichever occurred first.*

8. The terms "birth following labour" and "emergency CS" are both used. Assuming that they refer to the same thing, consistent terminology should be used throughout the manuscript.

RESPONSE: In the below sentence where we use the term “birth following labour” we used the term deliberately to refer to all births, regardless of mode of delivery, that included labour. This includes vaginal births, emergency caesarean sections, and elective caesarean sections performed before the scheduled date because of spontaneous labour.

PAGE 11, LINES 313-314: *A higher relative risk was observed when labour did not occur compared with births following labour (HR 1.12, 95% CI 1.12-1.13, p<0.001) (S2 Figure).*

**Reviewer #3:** Thank you for allowing me to review this manuscript.

Here, Miller and colleagues present the results from a large multicenter registry based study on the associations between CS delivery and childhood risk of hospitalizations with infectious cause in the first five years of life. The topic of research is not in itself novel, but never before has this association been shown in such a large cohort. I must congratulate the authors in collecting this huge number of women/children for analysis. The text is clearly written and the study is well performed, though some concerns do arise. The sub-analyses are also interesting to evaluate potential mechanisms and diverging associations.

1. CS delivery has time and time again been associated with asthma development in childhood. Asthma is almost always preceded by asthmatic episodes typically initiating in the first years of life and the number one cause of hospitalization in children. Asthmatic episodes are almost always triggered by viral or bacterial infections in this window. Could asthma be the underlying cause for the associations observed? I would like to see the associations adjusted for childhood asthma and stratified for hospitalizations with/without concurrent asthma/wheeze.

RESPONSE: We have included the suggested analysis. Using the Western Australia data, which were readily available to address this important issue and also have been validated, we looked at the association of mode of birth with the separate outcomes of a) infection-related hospitalisations with a concurrent diagnosis for asthma (J45)/wheeze (R06.2) and b) infection-related hospitalisations without a concurrent diagnosis for asthma/wheeze. The similar results for the separate outcomes support that the overall associations that we observe in our study are not solely due to asthma. We have added the following text and table to the manuscript:

PAGE 9, LINES 259-263: *Fourth, to consider if asthma, which may be associated with CS birth [26], was an underlying cause for the observed associations, we estimated the association between mode of birth and the separate outcomes of infection-related hospitalisation with and without a concurrent diagnosis for asthma and/or wheeze using the Western Australia data.*

PAGE 11, LINES 326-328: *Analyses restricted to infection-related hospitalisations either with or without a concurrent asthma and/or wheeze diagnosis were similar to the overall analyses that included all infection-related hospitalisations (S5 Table).*

PAGE 13, LINES 374-377: *We adjusted for concomitant asthma and/or wheeze, which has been repeatedly associated with CS birth [26], and which may contribute to the observed associations between birth mode and infections; findings were largely unchanged.*

**S5 Table: Sensitivity analysis – Hazard ratios for infection-related hospitalisations with and without a concurrent diagnosis of asthma and/or wheeze, Western Australia data**

|  | **Western Australia specific estimates** | | |
| --- | --- | --- | --- |
|  | **Infection-related hospitalisations with asthma/wheeze*** | **Infection-related hospitalisations without asthma/wheeze*** | **All infection-related hospitalisations** |
| **Mode of birth** | **Adjusted HR (95% CI)** | **Adjusted HR (95% CI)** | **Adjusted HR (95% CI)** |
| Vaginal | ref | ref | ref |
| Any caesarean section | 1.15 (1.10-1.21) | 1.12 (1.10-1.14) | 1.12 (1.10-1.13) |
| Emergency caesarean section | 1.17 (1.10-1.24) | 1.11 (1.08-1.13) | 1.11 (1.09-1.13) |
| Elective caesarean section | 1.14 (1.08-1.21) | 1.14 (1.12-1.16) | 1.13 (1.11-1.15) |

*All other infection-related hospitalisation excluded from the analyses.

Asthma was identified with ICD-10 code J45; wheeze was identified with ICD-10 code R06.2.

Estimates are from recurrent events models fitted for total time. Models adjusted for: sex, gestational age, birth weight z-score, smoking during pregnancy, maternal age at birth, parity, area level deprivation, birth year, medical indication for type of delivery, and season of birth.

2. Children born by CS are more often hospitalized right after birth and also more often treated for infections here. Was this potential hospital stay associated with the outcomes? E.g. the CS might only associate with later infections if the child were hospitalized after birth because of infections or other complications.

RESPONSE: Although children born by CS are hospitalized immediately following delivery for a few days, this is usually because their mothers have prolonged hospital stay post-operatively, rather than for infection-related indications in the infant. Indeed, immediate (i.e. early-onset, vertically acquired) neonatal sepsis may be *less* common in those born by CS. For example, guidelines for neonatal group B streptococcal (GBS) infection usually consider infants born by pre-labour CS to GBS colonised mothers as being a lower risk group. Additionally, the neonatal early-onset sepsis calculator algorithm tested many potential early onset sepsis risk factors and mode of birth was found to not be a key risk factor (PMID: 22025590, PMID: 23177799). For our study, follow-up began after the birth admission so any infections that occurred right after birth and before the child was discharged were not included in the analyses.

3. Microbiome hypothesis may be appealing, but other than differences between elective and emergency CS you have not presented much data to support this, so it comes off as speculative. To get closer to whether the associations might actually be caused by microbial derangements after CS, it would be a great strength to show that similar outcome associations existed among vaginal delivered children whose mothers were treated during birth.

RESPONSE: Assuming the reviewer is referring to prenatal antibiotic use, in the text, we reference our previous work (Miller et al, 2018) where we observed an association between prenatal antibiotic use and childhood infection-related hospitalisation, by mode of birth. In this previous analysis, among children followed until 5 years of age and born ≥36 weeks gestation, we observed an HR of 1.09 (95%CI: 1.05–1.13) in vaginally born children whose mothers had antibiotics during pregnancy compared to vaginally born children whose mothers did not have antibiotics pre, during or after pregnancy.

PAGE 8, LINES 251-254: *First, as we previously observed associations between prenatal antibiotic use, CS births and childhood infection-related hospitalisation risk, we used additional variables only available in the Danish data and adjusted overall models for antibiotic use three months prior to and/or during pregnancy [17].*

PAGE 14, LINES 377-379: *Finally, in the Danish population, where pregnancy antibiotic data were available, we adjusted for antibiotic exposure prior to and/or during pregnancy and the findings were unchanged (S4 Table).*

Miller JE, Wu C, Pedersen LH, de Klerk N, Olsen J, Burgner DP. Maternal antibiotic exposure during pregnancy and hospitalization with infection in offspring: a population-based cohort study. *Int J Epidemiol*. 2018;47(2):561-71.

4. A potential issue, when evaluating associations in such large datasets are the potential of insignificant significance. Would it be possible to calculate a population attributable risk fraction (PARF)?

RESPONSE: We calculated population attributable fractions for each site, included in the supporting information, and have expanded on its implication in the text. The following sections are in the text:

PAGE 9, LINES 272-273*: We calculated site-specific population attributable fractions to quantify the percentage and number of infection-related hospitalisations attributable to CSs.*

PAGE 12, LINES 340-344: *Population attributable fractions are provided in S8 Table. Two to three percent of children with infection-related hospitalisations could attribute their infection to being born by CS. Among the 1,502,537 children who had an infection-related hospitalisation in our study, about 14,000 calculated children had infections that could be attributed to being born by emergency CS and 18,500 to being born by elective CS.*

5. Maybe I missed it, but how did antibiotics to the mother in pregnancy affect the associations?

RESPONSE: Antibiotic data during pregnancy were not available for all of the jurisdictions. These data were available in Denmark and we did a sensitivity analysis where we adjusted for their use in the model. This is detailed in the manuscript on:

PAGE 8, LINES 251-254: *First, as we previously observed associations between prenatal antibiotic use, CS births and childhood infection-related hospitalisation risk, we used additional variables only available in the Danish data and adjusted overall models for antibiotic use three months prior to and/or during pregnancy [17].*

PAGE 14, LINES 377-379: *Finally, in the Danish population, where pregnancy antibiotic data were available, we adjusted for antibiotic exposure prior to and/or during pregnancy and the findings were unchanged (S4 Table).*

**S4 Table: Sensitivity analysis - prenatal antibiotic use and gestational age, Danish data**

|  | **All births** | **All births** | **Births ≥ 30 weeks** |
| --- | --- | --- | --- |
|  | **Fully adjusted** | **Fully adjusted  + prenatal antibiotic use** | **Fully adjusted** |
| **Mode of Birth** | **Hazard Ratio (95% CI)** | **Hazard Ratio (95% CI)** | **Hazard Ratio (95% CI)** |
| **Vaginal** | ref | ref | ref |
| **Any caesarean section** | 1.13 (1.11-1.14) | 1.12 (1.11-1.14) | 1.13(1.11-1.14) |
| **Emergency caesarean section** | 1.13 (1.11-1.15) | 1.12 (1.11-1.14) | 1.13 (1.11-1.14) |
| **Elective caesarean section** | 1.13 (1.11-1.15) | 1.12 (1.11-1.14) | 1.13 (1.11-1.15) |

Estimates are from 1st event models adjusted for: sex, gestational age, birth weight z-score, smoking during pregnancy, maternal age at birth, parity, area level deprivation, birth year, medical indication for type of delivery, and season of birth.

6. No line numbers in manuscript make specific comments less trivial...

RESPONSE: Sorry for this inconvenience, we have now added line numbers.

7. "gestational age (<28, 28-<32, 32-<34, 34-<36, 36-<38, 38-40, >40 weeks)", I would suggest more detailed i.e. weekly categorization around 37-40.

RESPONSE: We aimed to keep the GA intervals similar (2 weeks) where numbers allowed to allow for comparisons across the range of gestations. We agree that late preterm/early term is an important and often unrecognised determinant of infection-related and other outcomes and have previously published data highlighting this (PMID: 27052469). While there are other ways of presenting the data, for the current analysis, we believe that the current 2-week bands are the most informative overall.

8. A major difference between elective and emergency CS is that the birth induction is natural in most emergency sections, whereas in many countries elective is performed at 38+0. Gestational age is a major factor for perinatal outcomes - the two week difference may carry a large part of the effect.

RESPONSE: In our previous work (PMID: **27052469), we have used the usual reference group of 38-40 weeks GA. In the current study, vaginal deliveries, emergency and elective CS deliveries occur at around the same gestational age; for example, in Australia, elective CSs are performed at 39+ weeks and in Denmark at 38+3 to 39+3 with a clear tendency towards 39+3. Moreover, we adjusted for GA, so we believe that small variations in GA around term are unlikely to be driving the consistent associations observed.**

9. Since you are doing Cox regression I suggest you try various stratified Cox regression. a) stratified by year of birth; b) stratified by gestational age in detailed categories.

RESPONSE: In our model we adjust for year of birth and gestational age. We also did a subgroup analysis in children ≥37 gestational weeks. Please see below text from the Methods. We feel these approaches are sufficient to address our primary research questions.

PAGE 7, LINES 220-225:*Multivariable analyses included smoking during pregnancy (no/yes), maternal age at birth (<20, 20-<25, 25-<30, 30-<35, ≥35 years), parity (0, 1, 2, ≥3),* ***gestational age (<28, 28-<32, 32-<34, 34-<36, 36-<38, 38-40, >40 weeks),*** *birth weight (gestational age- and sex-specific z-score percentiles: ≤10, >10-25, >25-75, >75-90, >90), sex,* ***birth year****, season of birth (winter, spring, summer, autumn), socio-economic status (as described), recorded hypertensive disorders or diabetes mellitus during pregnancy (no/yes).*

PAGE 8, LINES 241-245: *Finally, each site estimated infection-related hospitalisation risk in a maternal sub-population considered at low risk for adverse outcomes, defined as cephalic-presenting infants born ≥37 weeks gestational age, with birth weight between the 10-90th percentiles for gestational age and sex, born to women aged 20-34 years without any reported hypertensive disorders or diabetes preceding or during pregnancy.*

10. How do you account for increasing rates of CS if you do not adjust for birth year? I suggest you stratify by birth year.

RESPONSE: With respect, we do adjust for birth year in 5 year intervals in all models. Please see below bolded text from the Methods.

PAGE 7, LINES 220-225:*Multivariable analyses included smoking during pregnancy (no/yes), maternal age at birth (<20, 20-<25, 25-<30, 30-<35, ≥35 years), parity (0, 1, 2, ≥3), gestational age (<28, 28-<32, 32-<34, 34-<36, 36-<38, 38-40, >40 weeks), birth weight (gestational age- and sex-specific z-score percentiles: ≤10, >10-25, >25-75, >75-90, >90), sex,* ***birth year,*** *season of birth (winter, spring, summer, autumn), socio-economic status (as described), recorded hypertensive disorders or diabetes mellitus during pregnancy (no/yes).*

11. The association between prenatal antibiotics and off-spring outcomes may be confounded (check out PMID: 25066330 also in Danish registry data showing the effect of prenatal antibiotics is not likely to be causal).

RESPONSE: Where data were available (from Denmark) we performed a sensitivity analysis to take into account potential confounding with further adjustment for maternal antibiotic use three months prior to and/or during pregnancy and the results were essentially unchanged. We further accounted for potential confounding by performing a subgroup analysis in a low risk population and by including a negative control analyses in the Western Australia population where the data were available and validated. Please see below section from the discussion:

PAGE 13, LINES 368-379: *In contrast to previous studies, we attempted to address potential confounding by a series of sensitivity analyses. We analysed a pre-defined sub-population considered at low obstetric risk in whom findings were similar to the overall cohort, indicating that confounding by indication was unlikely to be responsible for the observed associations. In addition, where data were available, we analysed a negative control outcome (hospitalisation with trauma) and observed no association with mode of birth, which gives confidence that our findings are not reflective of bias or unmeasured confounding. We adjusted for concomitant asthma and/or wheeze, which has been repeatedly associated with CS birth [26], and which may contribute to the observed associations between birth mode and infections; findings were largely unchanged. Finally, in the Danish population, where pregnancy antibiotic data were available, we adjusted for antibiotic exposure prior to and/or during pregnancy and the findings were unchanged (S4 Table).*

12. "When mode of birth and labour onset were jointly considered, the relative risks across exposure combinations, compared with vaginal births, were similar in size to the overall findings (Table S2)." → I do not agree. Your table S2 shows a similar difference in risk between pre/post labour as between elective/emergency CS in the Danish data.

RESPONSE: When elective or emergency CS were considered with or without labour, the effect sizes relative to vaginal delivery were essentially the same (the CIs overlap for all groups). These findings (ranging from 1.10-1.13) were similar to the overall findings. We have added the country-specific overall findings (underlined) to the table for comparison.

**S2 Table: Risk of infection-related hospitalisation by mode of birth and presence of labour**

|  | **Western Australia** | **Denmark** |
| --- | --- | --- |
| **Mode of Birth** | **Hazard Ratio (95% CI)** | **Hazard Ratio (95% CI)** |
| Vaginal birth | ref | ref |
| All Emergency Caesarean Sections | 1.11 (1.09-1.13) | 1.12 (1.10-1.13) |
| Emergency Caesarean Section no labour | 1.10 (1.06-1.14) | 1.13 (1.11-1.16) |
| Emergency Caesarean Section with labour | 1.11 (1.09-1.13) | 1.11 (1.09-1.13) |
| All Elective Caesarean Sections | 1.13 (1.11-1.15) | 1.13 (1.11-1.14) |
| Elective Caesarean Section no labour | 1.13 (1.11-1.15) | 1.13 (1.11-1.15) |
| Elective Caesarean Section with labour | N/A | 1.11 (1.05-1.14) |

Estimates not available for New South Wales (Australia), Scotland, and England. Estimates are from recurrent events models fitted for total time. Models adjusted for: sex, gestational age, birth weight z-score, smoking during pregnancy, maternal age at birth, parity, area level deprivation, birth year, medical indication for type of delivery, and season of birth.

13. In the Danish data you should be able to distinguish between induced and spontaneous delivery similar to the WA and not just from the CS coding. I don't believe your table S2 of DK vs WA are showing comparable data.

RESPONSE: For this study we did not differentiate between induced and spontaneous labour but rather if any labour occurred (yes/no). S1 Table illustrates the available data used to categorise births based on mode of birth and onset of labour. We feel that analyses into the type of labour would be better placed in a future study.

S1 Table – Variable definition

| **DENMARK** |  |  |
| --- | --- | --- |
| **Description** | **Variable** | **Data source** |
| **Caesarean section** |  |  |
| Emergency CS prelabour | KMCA10A | Medical Birth Register |
| Emergency CS in labour | KMCA10E | Medical Birth Register |
| Elective CS prelabour | KMCA10B | Medical Birth Register |
| Elective CS in labour | KMCA10D | Medical Birth Register |
| **WESTERN AUSTRALIA** |  |  |
| **Description** | **Variable** | **Data source** |
| **Caesearean section** |  |  |
| Emergency | Method of birth = 8 | Midwives Notification System |
| Elective | Method of birth = 7 | Midwives Notification System |
| **Onset of labour** |  |  |
| Yes | onset of labour = 1 "spontaneous" OR = 2 "induced" | Midwives Notification System |
| No | onset of labour = 3 "no labour" |  |

14. How is labour defined in DK in figure S2?

RESPONSE: Please refer to S1 Table illustrated in the previous response for the variable definitions. For S2 Figure, labour was defined in Denmark from the caesarean section variables (KMCA10A, KMCA10E, KMCA10B, KMCA10D). KMCA10A & B were categorised as births without labour, KMCA10E & D were categorised as births with labour. Please note, that the Danish coding practise has changed slightly after the study period (e.g. for certain types of breach CSs). All vaginal births were coded as births with labour.

**S2 Figure: Site-specific and meta-analysis hazard ratios for infection-related hospitalisation in births without labour**

Estimates are from recurrent events models fitted for total time. Models adjusted for: sex, gestational age, birth weight z-score, smoking during pregnancy, maternal age at birth, parity, area level deprivation, birth year, medical indication for type of delivery, and season of birth. Reference is births with labour. I-V: inverse-variance weighted fixed effects model, D+L: DerSimonian and Laird random effects model.

15. It seems to be just as large an effect of no-labour as of CS -- this does not lend support to your microbiome interpretation.

RESPONSE: The analysis shown in S2 Figure is comparing the risk of deliveries without labour (ie. exclusively elective CS) versus deliveries with labour (ie. vaginal delivery, most emergency CS and some elective CS). As expected, the adjusted HR approximates to that for elective CS in our overall analyses which used vaginal delivery as the reference (HR 1.13, 95% CI 1.12-1.13). With respect, we believe the reviewer may have misinterpreted this graph as it is essentially capturing the elective CS group.

16. Figure 3 is not well explained -- who is the low risk group? The reference group?

RESPONSE: Figure 3 are results from the maternal sub-population considered at low risk for adverse outcomes. The definition of the subgroup is explained in the methods section below and the reference is stated in the footnote of the figure.

Methods PAGE 8, LINES 241-245: *Finally, each site estimated infection-related hospitalisation risk in a maternal sub-population considered at low risk for adverse outcomes, defined as cephalic-presenting infants born ≥37 weeks gestational age, with birth weight between the 10-90th percentiles for gestational age and sex, born to women aged 20-34 years without any reported hypertensive disorders or diabetes preceding or during pregnancy.*

***Figure 3 - Site-specific and meta-analysis hazard ratios for infection-related hospitalisations in children born to a low risk population of mothers***

*Estimates are from recurrent events models fitted for total time. Models adjusted for: sex, gestational age, birth weight z-score, smoking during pregnancy, maternal age at birth, parity, area level deprivation, birth year, medical indication for type of delivery, and season of birth.* ***Reference is vaginal births****. I-V: inverse-variance weighted fixed effects model, D+L: DerSimonian and Laird random effects model. Low risk population of births defined as cephalic presenting infants born ≥37 weeks gestational age, with birth weight between the 10-90th percentiles for gestational age and sex, born to women aged 20–34 years without any reported medical conditions during pregnancy.*

17. In the discussion you mention the changes in policy - why not investigate the effects of these?

That could be very interesting and straight-forward in your data?

RESPONSE: We agree that this is interesting, but contend ascertaining with confidence whether changes in policy and guidelines actually result in individual-level changes in clinical practice across 5 jurisdictions is far from trivial. It was certainly beyond the scope of the current analysis.

18. Generally the whole discussion seems a bit too long and not on point with your data. Too much unfounded mechanistic talk. I would suggest more testing and discussion on the differences between countries? Could some of it be explained or elicited? As such it seems like your overall difference between elective/emergency CS is carried by England. This should be discussed in detail. And on the same line of thoughts, you see very similar estimates in DK: why?

RESPONSE: We have re-written the Discussion and reduced the length and prominence of the more speculative mechanistic interpretations that were not directly explored by the current study.

The elective CS results are consistent across jurisdictions and within the low risk sub-group analyses, the emergency CS results for England (HR 1.04, 1.03-1.05) and NSW (HR 1.05, 1.04-1.07) are consistent. This highlight the importance of our study – if single jurisdictions publish their results in isolation, then results may be interpreted differently/overconfidently. With our standardised analysis performed in multiple jurisdictions, we can be confident about the internal/external validity of the consistent findings and less confident about the less consistent findings. We cannot rule out a smaller difference in the Danish population but this may point to factors that modulate the potential effect such as choice of antibiotic, clinical practice, etc or it may be a chance finding.

19. Missing labour in table 1.

RESPONSE: We have added “Presence of Labour” to Table 1.

20. Small detail but some of the references background study are based on the same registry data that you are examining (ref 7).

RESPONSE: We are aware and are not trying to self-cite gratuitously. There are limited published population level data addressing some of the research questions and some of it is our previous work.

21. "population-specific obstetric practice may have unknown effects" -- could you not investigate this?

RESPONSE: Data related to obstetric practice were not available.


Reviewer #4: May 2020
Review- mode of birth and risk of infection releated hospitalization in childhood
Thank you for the opportunity to review this manuscript.
This study explores the prevalence of infection releated hospitalization in childhood according to delivery mode. Including over 7 million patients from four different countries. The HR was higher in the CS group for all infection related hospitalizations.

General remarks:
This is a overall interesting study with a large study size and a well thought out study protocol, there a re only a few remarks:
1. In the limitation section, please add that there is only record of hospitalizations and there is no data regarding infections which were treated ambulatory.

RESPONSE: Infection-related hospitalisations is a robust and widely accepted measure of infection severity and reflects hospital admissions due to infections of sufficient severity to warrant inpatient care and are less likely to be affected by social differences or health-seeking behaviour. We have expanded on its use in the text:

PAGE 14, LINES 383-387: *The outcome was infection-related hospitalisation, which minimises differences observed in primary care or emergency department presentations that may reflect social gradients in health literacy, health-seeking behaviour, or physician management rather than clinical severity [28].*

PAGE 15, LINES 410-413: *Finally, data on infections managed in primary care or in emergency departments and on postnatal factors that influence infection risk, such as infant feeding, vaccination status, and postnatal smoke exposure were unavailable.*

2. In the discussion you mention corticosteriod treatment as a part of the treatment protocol in all elective CS, is this true? If so, I would add that it is not a long term treatment.

RESPONSE: It was recommended practice in all jurisdictions, apart from Denmark, in the latter part of the study period to give antenatal corticosteroids to mothers delivering via CS to reduce infant respiratory morbidity. However we do not have data on corticosteroid exposure. We have added the underlined to the text:

PAGE 16, LINES 429-432: *Additional explanations include possible effects of short-term antenatal corticosteroids given to mothers delivering via CS to reduce infant respiratory morbidity, which was recommended practice in all jurisdictions (apart from Denmark) during the latter part of the study period.*

3. Sheiner et all published a very similar study last year, this study too had a large sample size, please refer to this study with similarities and differences. By the way, in his study population, corticosteriod population is not given automatically in all cases of elective CS.

RESPONSE: I believe the reviewer is referring to PMID: 30256312 where the study focuses on uncomplicated pregnancies and vaginal and elective caesarean births. This study reported a slightly higher risk for total infectious morbidity-related hospitalisations among children born by elective caesarean section than our study. The higher estimates may be related to neonatal infections, which did not seem to be excluded. We included the following text in the introduction and discussion:

PAGE 5, LINES 156-159: *A recent Israeli study among uncomplicated pregnancies and births (n = 138,910) estimated a 10% and 23% increased risk of hospitalisations with infection up to age 18 years among term only and preterm only elective caesarean section births, respectively [15].*

PAGE 13, LINES 364-369: *Our findings are in keeping with smaller population-level, single-jurisdiction studies that largely focus on specific infection outcomes, such as lower respiratory tract and gastrointestinal infections and which show that CS is associated with increased infection-related hospitalisation, and (where data are available) with greater risk following elective CS [11-15]. In contrast to previous studies, we attempted to address potential confounding by a series of sensitivity analyses.*
