# Supplementary material for: Mode of birth and risk of infection-related hospitalisation in childhood: A population cohort study of 7.17 million births from 4 high-income countries
Source: PLoS Med. 2020 Nov 19;17(11):e1003429. doi: 10.1371/journal.pmed.1003429 (PMC7676705; doi:10.1371/journal.pmed.1003429)
Supplement: Attachment — Submitted filename: Revisions_14Oct2020.docx [file pmed.1003429.s016.docx]

October 14th, 2020

PMEDICINE-D-20-00075R3

Dear PLOS Medicine,

I apologise for the oversight of not including the figures. All figures are now included in the submission, labelled correctly, and in .tif format. Thank you again for considering our paper for publication.

Sincerely,

Jessica Miller

1. We have noticed that you have cited Figs 1, 2, 3 & 4 in the manuscript file. However, there are no corresponding files uploaded to the submission. Please ensure that all files are present.

RESPONSE:
I apologise for this oversight (!). The figures are now included in the submission.

2. Further to the above request, please provide all main figures in .tif or .eps format. For more information about how to convert your figure files, please see our guidelines: [http://journals.plos.org/plosbiology/s/figures](https://protect-au.mimecast.com/s/P5BtCXLWLNFnE9wKs9pm4A?domain=journals.plos.org). Once you've converted your files to .tif or .eps, please also make sure that your figures meet our format requirements: [http://journals.plos.org/plosbiology/s/figures#loc-file-requirements](https://protect-au.mimecast.com/s/3bGYCYW8WOT3V9rlc3ykk1?domain=journals.plos.org) / [https://journals.plos.org/plosmedicine/s/figures#loc-how-to-submit-figures-and-captions](https://protect-au.mimecast.com/s/07eMCZY1YPtMKjJgcNfhfO?domain=journals.plos.org)

RESPONSE:

The figures are all in .tif format.

3. Further to the above request, please ensure that all main figures are referenced in the manuscript as Fig 1, Fig 2, etc. (including capitalization), rather than Figure 1, Figure 2, etc. Please note, however, that the file names themselves must not include the space, i.e. Fig1.tif, Fig2.tif. Please ensure that all main figure files are clearly labeled as such in the File Inventory.

RESPONSE:
I have checked the manuscript and all figures are referenced as Fig 1, Fig 2, etc. and the file names are Fig1.tig, Fig2.tif etc. The only mention of “Figure” is in “Figure Legend”.
